# Antibacterial and Anti-Biofilm In Vitro Activities of Essential Oils Against Bovine Mastitis-Related Pathogens

**DOI:** 10.3390/antibiotics15101038

**Published:** 2026-10-19

**Authors:** Alice Caneschi, Donatella Scarafile, Monica Modesto, Maurizio Scozzoli, Paola Mattarelli, Anna Zaghini

**Affiliations:** 1 SIROE—Italian Society for Research on Essential Oils (Società Italiana per la Ricerca sugli Oli Essenziali), Viale Regina Elena 299, 00161 Roma, Italy; maurizio@greenvet.com; 2 Department of Agricultural and Food Sciences, University of Bologna, Viale G. Fanin 44, 40127 Bologna, Italy; donatella.scarafile2@unibo.it (D.S.); monica.modesto@unibo.it (M.M.); paola.mattarelli@unibo.it (P.M.); 3 Department of Veterinary Medical Sciences, University of Bologna, Via Tolara di Sopra 50, Ozzano Emilia, 40064 Bologna, Italy; anna.zaghini@unibo.it

**Keywords:** essential oils, bovine mastitis, antibacterial activity, antibiofilm activity, antimicrobial resistance (AMR)

## Abstract

**Background**: The inflammation of the mammary gland, known as bovine mastitis, is a major challenge for the dairy cattle industry globally. The main organisms responsible for mastitis are bacteria, classified as either contagious, such as *Staphylococcus aureus*, *Streptococcus dysgalactiae*, and *Streptococcus agalactiae*, or environmental, primarily *Streptococcus* spp. (e.g., *Streptococcus uberis*) and coliform species. Even though the emergence of antibiotic-resistant strains of pathogens poses a serious threat, antibiotics are still the primary treatment for mastitis. It is therefore necessary to explore alternative therapies, such as essential oils (EOs). This in vitro research intended to evaluate the efficacy of EOs against pathogens associated with bovine mastitis, as an alternative or complementary treatment to conventional antibiotics. **Methods**: The bactericidal activity of eleven essential oils and two blends was tested on five strains related to bovine mastitis: *S. agalactiae*, *S. dysgalactiae*, *S. uberis*, *S. aureus*, and *Staphylococcus epidermidis*. An assessment was carried out of both the reduction of biofilm formation and the inhibition of pre-formed biofilm against biofilm-formative strains of *S. aureus* and *S. epidermidis*. **Results**: *Origanum vulgare* and *Cinnamomum verum* resulted to be the EOs with the highest activity against the above-mentioned bacteria, inhibiting both bacterial growth and biofilm formation. **Conclusions**: This study emphasizes the relevance of investigating natural products and supports the hypothesis of appropriate use of EOs to reduce bacterial infections and provide an effective alternative to antibiotics in complementary therapy. Moreover, to avoid treatment failure, the combined use of available antibiotics and natural compounds should also be considered.

## 1. Introduction

Bovine mastitis is an inflammatory condition affecting the parenchyma of the mammary gland caused by microbial infections or physical trauma, resulting in alterations in the bacteriological, physical, and chemical characteristics of milk [1]. Because of reduced milk quality and production, cattle mastitis ranks as the leading cause of economic losses and represents one of the primary concerns for the dairy industry worldwide [2,3,4]. Bovine mastitis results from a combination of factors related to the animal, the environment, and pathogens; it can be divided into three classes depending on the severity of inflammation: clinical, subclinical, and chronic mastitis [5,6]. Clinical mastitis is obvious and easily identifiable due to noticeable milk or udder abnormalities and common symptoms (fever and loss of appetite) [7]. In subclinical mastitis, instead, no obvious signs are observed, but there is a reduction in milk production and an alteration in its composition [8]. Chronic mastitis refers to inflammation that persists for months, accompanied by clinical relapses.

Organisms such as bacteria, fungi and viruses invading the teat canal are considered the main cause of bovine mastitis [9]. Bacteria are the most common organisms responsible for mastitis and, depending on their origin, the infection can be divided into two classes: contagious and environmental [10]. Contagious pathogens, such as *Staphylococcus aureus, Mycoplasma* spp., and *Streptococcus agalactiae*, usually reside on the udder and the teat, colonizing and proliferating within the mammary duct [11]. This can lead to subclinical infections, usually accompanied by an increase in somatic cell count (SCC). Environmental pathogens, on the other hand, do not belong to the mammary gland microbiota; once inside the mammary gland, they reproduce, trigger the host immune response and are eliminated quickly [12]. The environmental pathogens mainly include coliform species (e.g., *E. coli*, *Klebsiella* spp., *Enterobacter* spp.), *Streptococcus* spp. (e.g., *Streptococcus uberis*), *Pseudomonas* spp., etc [13].

Bacteria responsible for mastitis, such as *S. uberis* and *S. aureus*, can also form biofilm [14]. Biofilm matrices consist of microbial cells, polysaccharides, water and other extracellular products, providing a protective environment ideal for bacterial proliferation [15]. Consequently, bacteria become more resistant to antibiotics and host defences. Therefore, the production of biofilm may result in challenges when treating recurrent infections [16], allowing bacteria to adapt to adverse environments [17].

A prompt and precise identification of the pathogens responsible, given their wide range, is essential both for controlling the spread of the disease (contagious organism) and for the correct selection of antibiotics for therapeutic purposes [7].

Antibiotics, such as penicillin, ampicillin, tetracycline, gentamicin, etc., are the main treatment for mastitis; these can be administered intramammary, intramuscularly or via intravenous infusion [18]. Ideal treatment duration should be long enough to clear subclinical mastitis yet short enough to prevent antibiotic resistance; the best time to treat mastitis is the dry period, with no milk production [19]. Despite antibiotics still being the main therapeutic approach, their effectiveness is limited, and bacterial clearance is often less than 60% [20], with the additional challenge of the emergence of antibiotic-resistant strains becoming a major issue in antibiotic treatment [21].

In order to prevent the spread of antimicrobial resistance, the Regulation (EU) 2019/6 of the European Parliament and of the Council of 11 December 2018 on veterinary medicinal products and repealing Directive 2001/82/EC, stipulates that, apart from exceptional cases, antimicrobial medicinal products shall not be used for prophylactic purposes [22].

The ability of microorganisms to survive or proliferate in the presence of a concentration of an antimicrobial agent (i.e., an antibiotic) normally effective in inhibiting or killing microorganisms of the same species is defined as antimicrobial resistance (AMR) [22]. Systematic use of antibiotics exerts selective pressure on both commensal bacteria, like the gut or skin microbiota, and pathogenic bacteria, which can lead to the dominance of antibiotic-resistant bacteria and the rise of resistant genes. Nowadays, AMR has a serious impact on society, both economically and in terms of public health [23], being a widespread and increasing phenomenon, and representing one of the most difficult global challenges.

AMR is far more complex in animals than in humans, and demands an even more careful and aware use of antibiotics [23]. The administration of antibiotics to food-producing animals, in particular, can leave residues in milk and in edible tissues [24]. It is therefore necessary to seek alternatives based on natural products such as essential oils obtained from medicinal plants, particularly when dealing with multidrug-resistant bacteria [25].

Plant extracts, including essential oils (EOs), have a long history of use in traditional medicine across the world, and their application in the treatment of bovine mastitis is of growing interest [26]. Several EOs with various biological properties are already being used in this area, although few in vivo studies report on their efficacy, pharmacokinetics and pharmacodynamics [27]. EOs’ antibacterial effects result from the loss of ions and the leakage of cellular components, caused by increased membrane permeability, reduced membrane potential, ATP depletion and disruption of proton pumps, resulting in cell lysis and cell death [28,29,30]. It is harder for microorganisms to develop resistance to essential oils, as they contain a wide variety of components. Furthermore, essential oils can exert antibacterial activity on both resistant and non-resistant pathogens [31,32]. Essential oils and their derived components have attracted growing interest, in recent years, thanks to their encouraging biological activities, as potential candidates for combating bacterial infections and reducing AMR [33,34,35]. Hence, it is crucial to understand the mechanism underlying the phytochemical action through rigorous experimental investigations for the development of new antimicrobial agents [36].

Given these promising results, this research aimed at evaluating the opportunity to use essential oils in the treatment of bovine mastitis as a potential alternative to conventional antibiotics. Essential oils, thanks to their additional antioxidant and antinflammatory capabilities, would also provide additional beneficial effects in the inflammatory status of bovine mastitis.

The in vitro bactericidal activity of eleven essential oils and two blends was investigated against five strains associated with bovine mastitis, *Streptococcus agalactiae*, *Streptococcus dysgalactiae*, *Streptococcus uberis*, *Staphylococcus aureus* and *Staphylococcus epidermidis*. The anti-biofilm activity against biofilm-formative strains of *Staphylococcus aureus* and *Staphylococcus epidermidis* was also assessed.

## 2. Results and Discussion

### 2.1. Chemical Characterization of Essential Oil (GC-FID Analysis)

The chemical characterizations of each EO considered in the present study are reported in the Supplementary Materials, in Tables S1–S13. Compounds are ranked by elution order, and the values are expressed as the percentage peak area relative to the total composition of each EO, as determined by GC-FID analysis.

### 2.2. Minimum Inhibitory Concentration (MIC) and Minimum Bactericidal Concentration (MBC)

The results of the antimicrobial screening (MIC and MBC) of the eleven essential oils and the two blends are given in Table 1, by comparing them for the various microorganisms (*S. agalactiae*, *S. dysgalactiae*, *S. uberis*, *S. aureus* and *S. epidermidis*).

The MIC values of the essential oils varied between 0.001% and >3.125% *v*/*v* (Table 1). In general, the effect was greater against bacteria of the genus *Streptococcus* than against those of the genus *Staphylococcus. S. dysgalactiae* was the most sensitive strain to the treatment with the EOs, while *S. epidermidis* was the most resistant one, being sensitive just to *Cinnamomum verum* (J.Presl), at the tested concentrations (from 3.125% to 0.001% *v*/*v*).

The EOs of *Origanum vulgare* (L.), *Cinnamomum verum*, *Thymus vulgaris* (L.), and Blend BR proved to be the most effective against *S. agalactiae*, *S. dysgalactiae* and *S. Uberis*, with MIC and MBC values ranging from 0.001% to 0.390% *v*/*v*, and from 0.001% to 0.780% *v*/*v*, respectively. Against *S. aureus* and *S. epidermidis*, the lowest MICs occurred with *C. verum*: 0.098% and 0.195% *v*/*v*, respectively. Among the EOs, *Eucalyptus globulus* (Labill.) and *Citrus limon* ((L.) Osbeck) exhibited the lowest antibacterial activity towards all pathogens; MBC resulted >3.125% *v*/*v*.

*Citrus limon* showed no antibacterial activity, despite the presence of limonene (57,55%) that showed antibacterial activity against *S. uberis* [37]. Limonene, indeed, such as thymol, can alter the composition of the fatty acids in cell membranes, while also affecting their respiration and permeability [38].

1.8-cineole, the main component (93.10%) of *Eucalyptus globulus*, is able to disintegrate the cell membrane and reduce and concentrate the cytoplasm, causing damage to the structure of *S. aureus* [33]. Nevertheless, the EO of *E. globulus* did not demonstrate an antibacterial property against the tested strains.

The composition of the EO of *Melaleuca leucadendra* (L.) highlighted as a major component the 1,8-cineole (67.85%). As for *Eucalyptus globulus*, this EO showed moderate activity just against *S. dysgalactiae* (0.780% *v*/*v*), suggesting that the destabilization in the membrane induced by 1,8-cineole might not be enough, alone, to inhibit the growth of the tested strains. It has been demonstrated in two studies that an essential oil from the *Melaleuca* genus, rich in 1,8-cineole, has a synergistic effect with rifaximin [39] and erythromycin [33] against *S. aureus.*

*Melaleuca alternifolia* (Cheel), known as tea tree oil (TTO), showed weak activity against *S. agalactiae* (1.560% *v*/*v*), moderate activity against *S. dysgalactiae* and *S. uberis* (0.780% *v*/*v*), and it was not active against the strains of *Staphylococcus* spp. at the tested concentrations (>3.125% *v*/*v*). In total, 80–90% of the oil is represented by the monoterpenes terpinen-4-ol, α-terpinene, 1,8-cineole, p-cymene, α-terpineol, α-pinene, terpinolene, limonene, and sabinene. The most abundant (41.21%) of these is terpinen-4-ol, which contributes significantly to the antimicrobial properties of the oil [40].

These results do not contradict the data reported from previous studies on the antibacterial effect of TTO against *Streptococcus* spp. and *Staphylococcus* spp., since the concentrations tested in our study are lower [41,42].

*Salvia rosmarinus* (Spenn.) showed good antibacterial activity against *S. dysgalactiae* (0.195% *v*/*v*). The EO has both antimicrobial and antioxidant attributes, which are primarily associated with monoterpenes, like 1,8-cineole (34.29%), pinene (33.84%), camphor (19.35%), borneol, limonene, camphene, and myrcene [43]. Contrary to the results of [44], in our study, *S. rosmarinus* did not appear to be active against most of the pathogenic bacteria.

Menthol (36.43%) is a major component of *Mentha × piperita* L. (peppermint), playing a role in the herb’s antioxidant and antimicrobial effects [45]. *M.* x *piperita* EO exhibited low MIC values against the *Streptococcus* strains, while it was not active against the *Staphylococcus* strains, confirming the results obtained by Tomanić et al. (2022) [46], who highlighted higher MIC values of the EO against *Staphylococcus* spp. compared to *Streptococcus* spp.

Both the EOs of *Lavandula angustifolia* (Mill.) and *Lavandula × intermedia* (Emeric ex Loisel.) showed a composition rich in linalool (37.32% and 33.33%, respectively) and linalyl acetate (30.64% and 32.20%, respectively). These two predominant compounds are considered responsible for the antimicrobial activity of these plants [47].

*Thymus vulgaris* was very effective against the strains belonging to the genus *Streptococcus*, with MIC values from 0.195% *v*/*v* to 0.390% *v*/*v*. The importance of the high presence of thymol (46.41%) and p-cymene (19.89%), within the essential oils, has been confirmed for the antibacterial properties of the genus *Thymus* [48,49,50]. Both thymol and carvacrol exert their antimicrobial activity by inducing changes in the structure and function of the cytoplasmic membrane that affect both the external and internal membranes. Thymol may additionally interfere with membrane proteins and intracellular targets. This interaction alters the membrane’s permeability and leads to the release of K^+^ ions and ATP [51]. Thymol has been shown to be highly effective against isolates from mastitis cows [41,52,53,54].

*Origanum vulgare* and *C. verum* were the only essential oils to be active against *S. aureus*. In many studies *O. vulgare* has been shown to be active toward major mastitis pathogens such as *S. aureus*, *E. coli*, *Streptococcus* spp., *S. dysgalactiae*, and *S. uberis*, along with methicillin-resistant *Staphylococcus aureus* (MRSA) [54,55,56]. The phenolic monoterpenoid carvacrol (79.29%) affects microbial cells by causing damage to the structure and function of their membranes, leading to enhanced membrane fluidity and permeability [57].

*C. verum* EO resulted the most active among the tested EOs. The EO was effective against all the tested strains, especially against *S. agalactiae* and *S. uberis*, with MIC values of 0.050% *v*/*v* and 0.001% *v*/*v* respectively (Table 1). This antibacterial property could be attributed to its characteristic secondary metabolites such as trans-cinnamaldehyde (79.03%), cinnamyl acetate, caryophyllene oxide, and β-caryophyllene [58]. These results agree with the literature in vitro studies that demonstrates the bactericidal effects of *Cinnamomum* EO against bovine mastitis pathogenic isolates [59,60], especially against Gram-positive bacteria [61].

Therefore, *O. vulgare* and *C. verum* may be regarded as promising candidates in the development of antibacterial treatments for antibiotic-resistant bacterial infections.

### 2.3. Biofilm

The anti-biofilm assay was carried out against a strain of *Staphylococcus aureus* (ATCC 6538) and a strain of *Staphylococcus epidermidis* (DSMZ 20044). The assays were conducted to evaluate the efficacy of the essential oils both in preventing cell attachment (anti-adhesion), at 0 h (effect on bacterial adhesion, EBA), and in inhibiting the growth of pre-formed biofilms, at 24 h (effect on pre-formed biofilm, EPB). The crystal violet assay quantifies the entire biofilm biomass, including extracellular polymeric substances formed during biofilm growth that bind or attach to the bacterial cells.

The result regarding the percentage (%) of biofilm inhibition of the three different tested concentrations (0.78, 0.39 and 0.195% *v*/*v*, μL/mL) of each essential oil are reported in the Supplementary Materials, in Figures S1–S20. The effects of the seven EOs and the two blends on the development and adhesion of biofilms of the tested mastitis-related agents are illustrated in Figure 1, Figure 2, Figure 3 and Figure 4. For comparison purposes, anti-biofilm activity was operationally classified as low (<25% biofilm reduction), moderate (25–50%), high (>50–75%), or very high (>75%); these author-defined categories do not represent internationally validated thresholds.

*C. verum* EO revealed a very good activity against the two strains, on both EBA and EPB, being the most effective. The strong antibacterial and anti-biofilm properties of cinnamon oil are definitely due to its high cinnamaldehyde level, as shown by Budri et al. (2015) [59], who found that cinnamaldehyde had a lower MIC than *Cinnamomum verum*, and it greatly decreased biofilm formation. *C. verum* was further evaluated against biofilm formation by *S. agalactiae*, where the EO was able to exert potent inhibitory activity [62].

*S. aureus* and *S. epidermidis* are two well-known biofilm producers and are commonly associated with intramammary infections, leading to chronic bovine mastitis. It is well established that staphylococcal isolates are the primary cause of biofilm-associated infections [63]. Specifically, the formation of biofilms by *S. aureus* strains and their multidrug-resistance profile increase the risk of chemotherapeutic failure [64].

The anti-biofilm effect of different essential oils against pathogenic isolates from bovine mastitis has been extensively studied, evaluating both the reduction in biofilm formation and the inhibition of pre-formed biofilm [37,59,65,66,67,68,69].

However, the published literature showed no prior research on the anti-biofilm potential of *L. angustifolia*, *L. × intermedia*, *M.* x *piperita*, *O. vulgare* and *T. vulgaris* against bovine mastitis-associated organisms.

**Figure 1:**
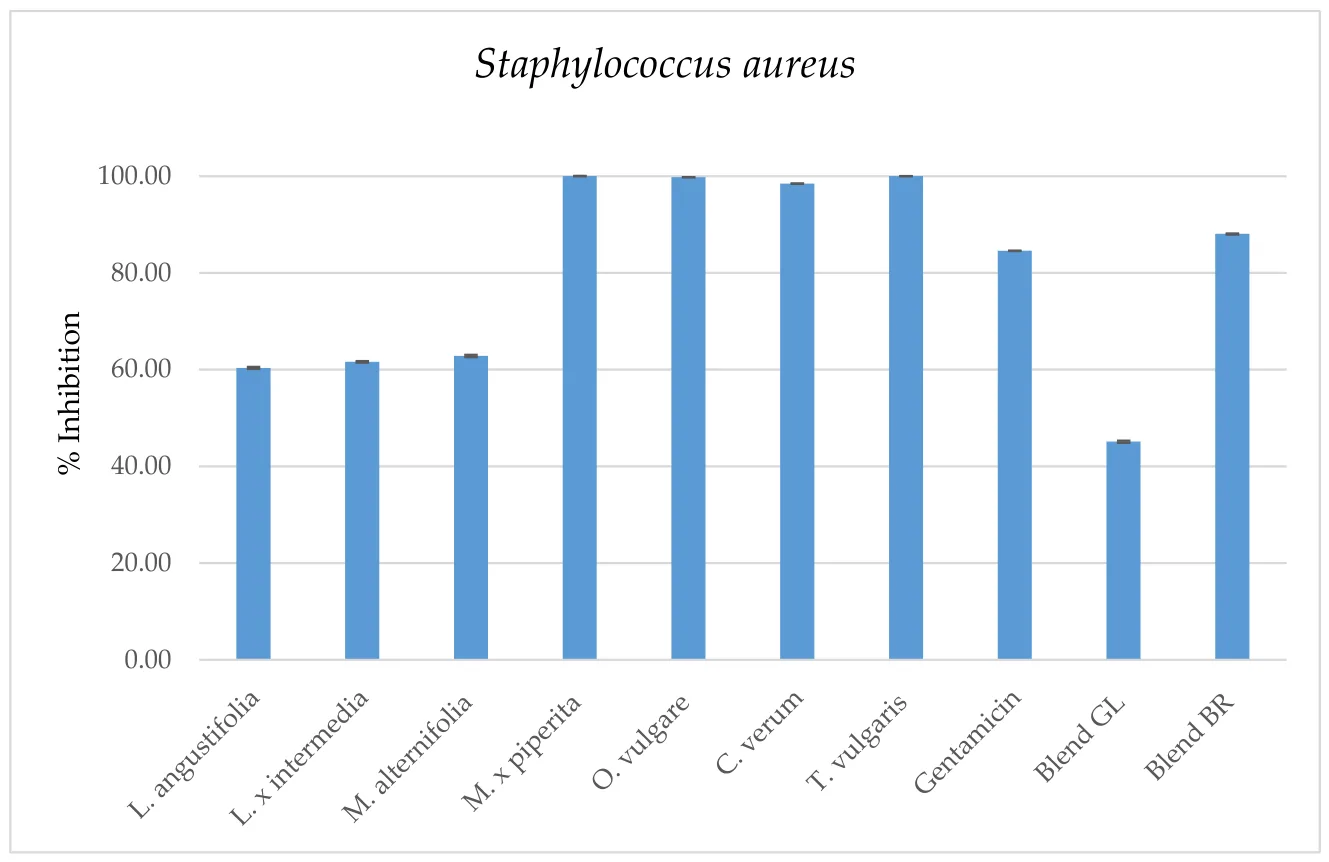
Effect on bacterial adhesion, EBA. Percentage (%) of biofilm inhibition of seven essential oils and two blends at a concentration of 0.39% *v*/*v* on *S. aureus*. Control with gentamicin at 1mg/mL. Vertical bars indicate ± standard deviation.

**Figure 2:**
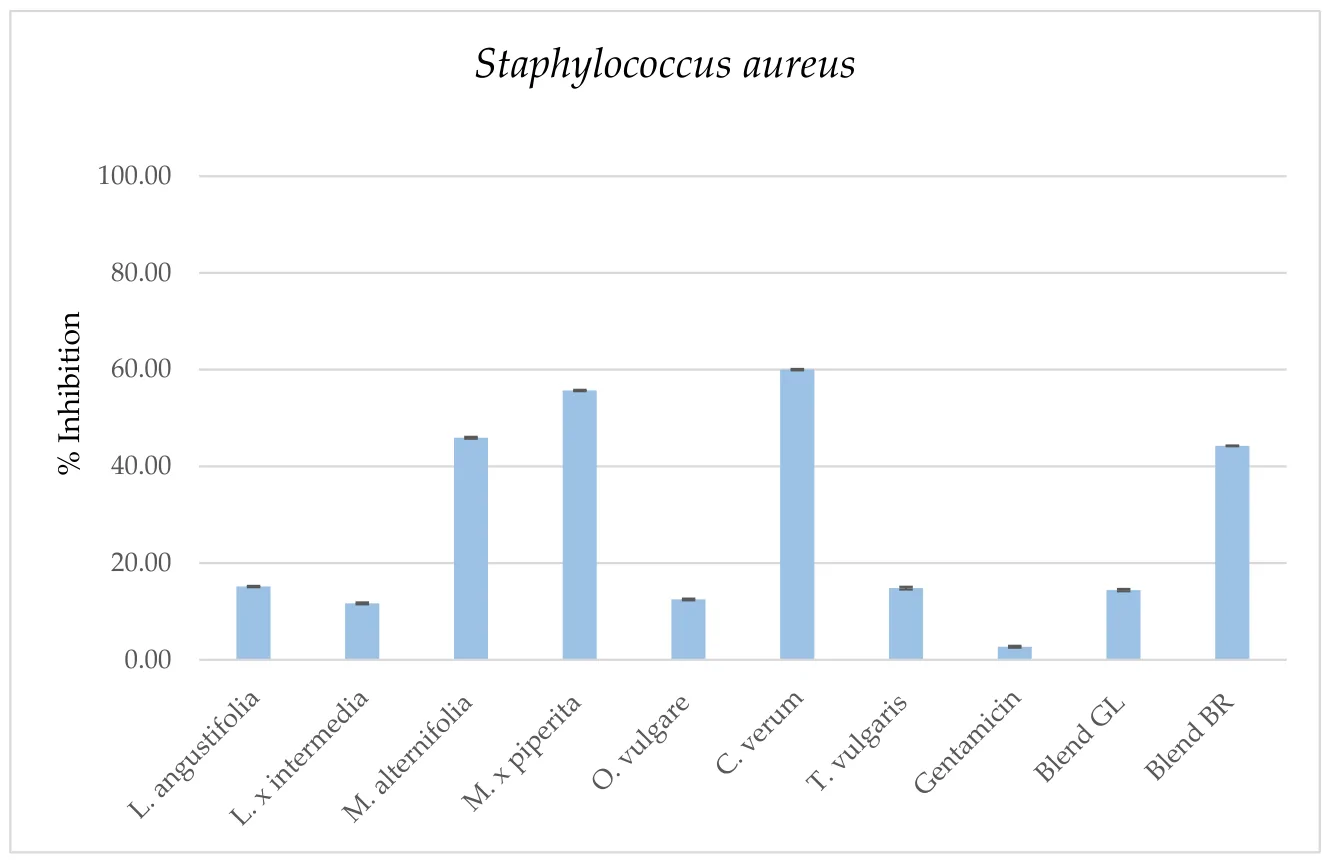
Effect on pre-formed biofilm, EPB. Percentage (%) of biofilm inhibition of seven essential oils and two blends at a concentration of 0.39% *v*/*v* on *S. aureus*. Control with gentamicin at 1mg/mL. Vertical bars indicate ± standard deviation.

**Figure 3:**
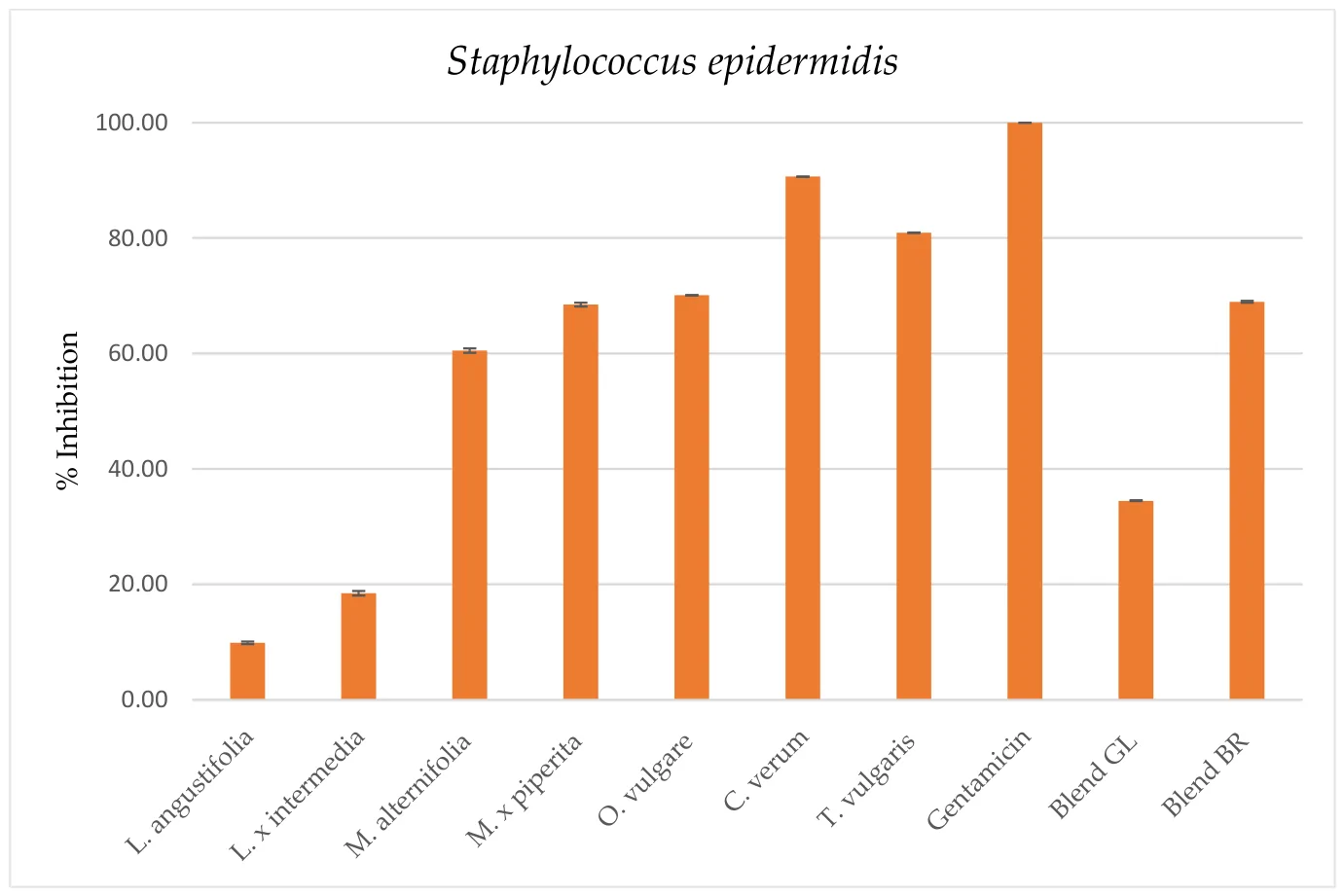
Effect on bacterial adhesion, EBA. Percentage (%) of biofilm inhibition of seven essential oils and two blends at a concentration of 0.39% *v*/*v* on *S. epidermidis*. Control with gentamicin at 1mg/mL. Vertical bars indicate ± standard deviation.

**Figure 4:**
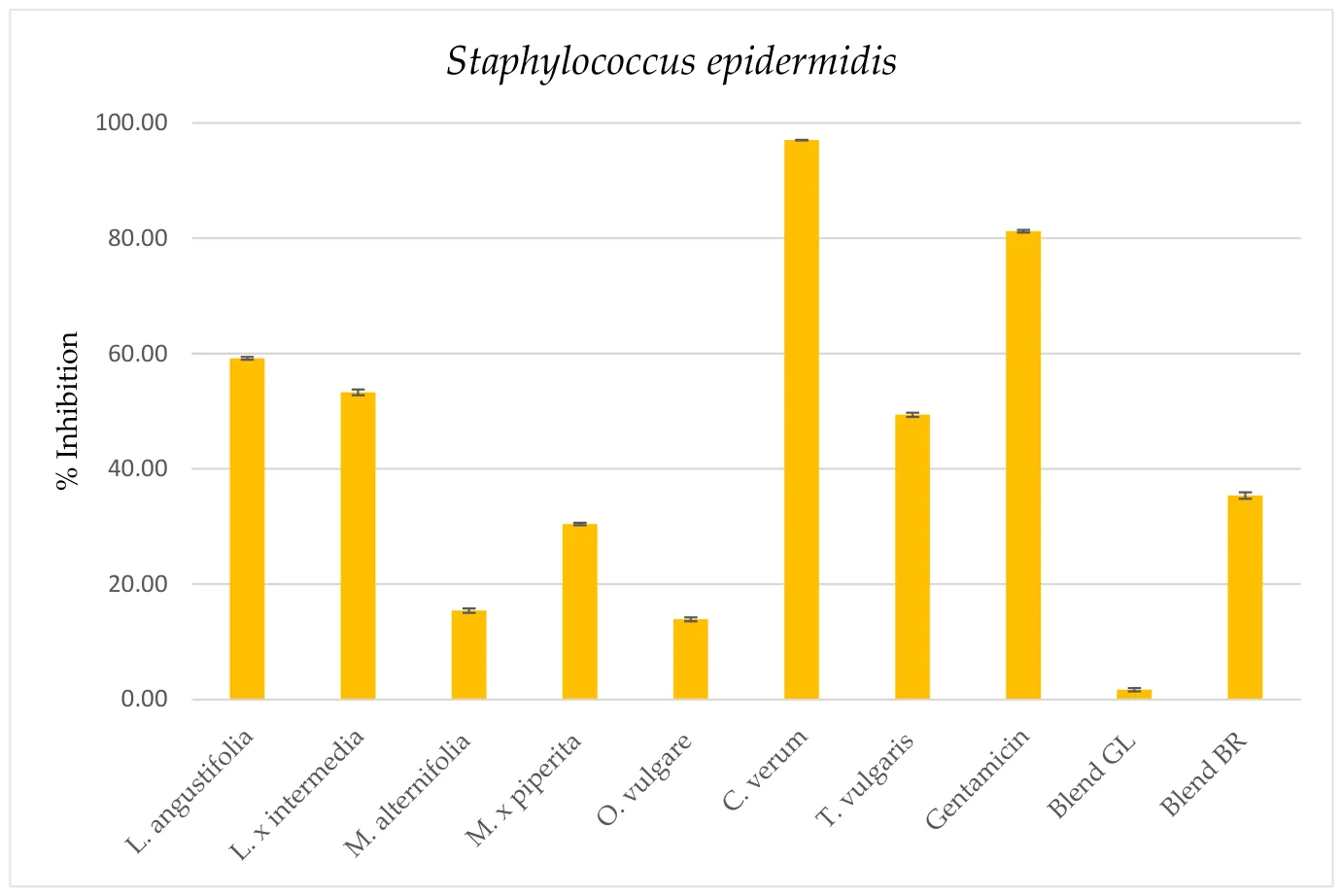
Effect on pre-formed biofilm, EPB. Percentage (%) of biofilm inhibition of seven essential oils and two blends at a concentration of 0.39% *v*/*v* on *S. epidermidis*. Control with gentamicin at 1mg/mL. Vertical bars indicate ± standard deviation.

In this study on bacterial adhesion (EBA), at a concentration of 0.39% *v*/*v*, all essential oils showed high inhibition of biofilm formation, above 50%, against the tested organism *S. aureus*, except the Blend GL (45.1%) (Figure 1). Indeed, most of the EOs had good to moderate inhibition on biofilm formation (EBA) against the strains tested. In general, the extracts were more effective at preventing biofilm formation (with higher activity on EBA, Figure 1 and Figure 3) than at destroying pre-formed biofilm (EPB, Figure 2 and Figure 4).

*M.* x *piperita*, *O. vulgare*, *C. verum* and *T. vulgaris* essential oils had the best biofilm inhibition, ranging from 97.67% to 100.00%, on EBA for *S. aureus*.

All tested essential oils, except for *L. angustifolia* and *L. × intermedia*, and Blend GL exerted high activity in the reduction (>50%) of development of biofilm, EBA, against *S. epidermidis.* The best activity was showed by *T. vulgaris* and *C. verum*, with inhibition values of 80.91% and 90.66% respectively.

On pre-formed biofilm, EPB, Blend GL, and essential oils of *O. vulgare* and *M. alternifolia* had low anti-biofilm activity (1.70% to 15.40%) against *S. epidermidis*, whereas Blend BR and EO of *M.* x *piperita* had moderate activity (31.67% to 35.38%).

Blend GL resulted to be less active against both the tested strains compared to the majority of the essential oil, while Blend BR demonstrated good activity, especially against *S. aureus*, even higher than the gentamicin control. These findings show how essential oils, mixed together, can have combined effects with each other, resulting in mixtures with more or less antibacterial activity.

The lowest concentration (0.195% *v*/*v*) of *L. angustifolia* essential oils increased biofilm growth of *S. aureus* in EPB, as well as the essential of *M. alternifolia* and Blend GL enhanced the growth of pre-formed biofilm of *S. epidermidis*. These results find support in some studies where an increase in biofilm growth has been shown after the treatment of pre-formed biofilm with both EOs and their components [70,71].

Indeed, after exposure to low concentration of *Origanum vulgare* EO, an increasing number of sessile cells in mature biofilm of *S. aureus* has been observed [72]. According to the authors, the stimulating effect of the oil occurs at concentrations of phenolic compounds below the MIC, as a reaction to stress conditions, thereby enhancing the ability of the microorganism to form biofilms [73].

Overall, this study revealed that essential oils from *O. vulgare* and *C. verum* exhibited the strongest activity against bacteria and biofilms. Our results are consistent with the literature, as various classes of bioactive secondary metabolites, like carvacrol and trans-cinnamaldehyde, terpenoids and flavonoids have been isolated from these species [57,58]. Given the pharmacological potential of trans-cinnamaldehyde and carvacrol, these molecules and other classes of metabolites contained in the EOs of *C. verum* and *O. vulgare* might be responsible for the observed activity. These findings are consistent with previously published results regarding the antimicrobial and anti-biofilm activities of *Cinnamomum* EO [59,62].

Several authors have suggested that treatment failure due to antimicrobial resistance could be addressed by combining available antibiotics with other natural products, such as EOs [67,74,75,76].

The current scenario of antimicrobial, and especially antibiotic, resistance, is indeed extremely worrying and the scientific community is investing time and effort in finding new solutions. Veterinary medicine, and farm animals in particular, have always been considered among the major players in the development of resistance due to the large-scale, and sometimes inappropriate, use of antibiotics. Such molecules are crucial in the treatment of specific diseases, so it is extremely important to “preserve” their effectiveness as much as possible. Moreover, the new European Union Regulations greatly reduce the possibility of preventive antibiotic use in animals and extremely limit the antibiotic categories allowed in veterinary medicine [22,77,78]. For this reason, it is necessary to find alternatives, especially in the treatment of those diseases caused by resistant bacteria.

## 3. Materials and Methods

### 3.1. Essential Oils

Eleven single essential oils and two blends were used for the study. In particular, *Origanum vulgare* (aerial parts), *Melaleuca alternifolia* (leaves), *Melaleuca leucadendra* (leaves), *Eucalyptus globulus* (leaves), *Mentha × piperita* (leaves), *Lavandula angustifolia* (flowers), *Lavandula × intermedia* (flowers), *Cinnamomum verum* (bark), *Salvia rosmarinus* (aerial parts), *Citrus limon* (peel), *Thymus vulgaris* (aerial parts), and two blends, GL and BR, confidential solutions under patent processing, containing mixtures of the above-mentioned EOs (Blend GL includes *E. globulus*, *O. vulgare*, *L. angustifolia*, *M. leucadendra*, *M. alternifolia*, *M. x piperita*, and *T. vulgaris.* Blend BR includes *M. alternifolia*, *O. vulgare*, *T. vulgaris*, *C. verum*, *M. leucadendra*, and *L. x intermedia*). All EOs and the blends were used as emulsions, dispersed in the surfactant Alkamuls (Glyceryl polyethyleneglycol ricinoleate, CEE additive cod. E484), and they were provided by APA-CT (Forlì, FC, Italy).

### 3.2. Chemical Characterization of Essential Oil (GC-FID Analysis)

The chemical analysis of all the EOs were obtained from the suppliers (Tables S1–S13). The chromatographic characterization of EOs was carried out using a gas chromatograph (GC) with a flame ionization detector (FID), following the protocol validated by Truzzi et al. (2025) [79].

Qualitative analysis and the semi-quantitative characterization of EOs were performed using an Agilent Technologies (Agilent Technologies, Milan, Italy) HP-5 cross-linked 5% phenyl–95% methyl polysiloxane (25 m × 0.2 mm i.d., 0.5 μm film thickness) capillary column on a 7820 gas chromatograph coupled with FID.

Compounds were recognized by matching the retention times of the chromatographic peaks with those of authentic reference standards analyzed under identical conditions and by matching the linear retention indices (LRIs).

Semi-quantitative data were derived from the mean of two analyses and calculated as the relative percentage amount of each analyte; specifically, the values were reported as the percentage peak area relative in comparison to the overall composition of each EO, as determined by GC-FID analysis.

### 3.3. Essential Oils Emulsions

The EOs emulsions were created using Alkamus, a surfactant (Glyceryl polyethyleneglycol ricinoleate cod. E484), approved for use in animal feed, as follows. The stock emulsion was prepared with the EO and Alkamus, in a proportion of 1:3 *v*/*v*; both were placed in an Eppendorf tube and vortexed for approx. 1 min; then, the growth medium, Muller Hinton (MH), or Brain Heart Infusion (BHI), was added and vortexed again for at least 3 min, after which a milky to opaque emulsion was obtained. Subsequently the stock solution was diluted with growth medium obtaining a concentration of EO of 6.25% *v*/*v*.

### 3.4. Microdilution Assay

Antibacterial activity of each of the essential oils and the two blends was assayed against *Streptococcus agalactiae* (DSMZ 2134), *Streptococcus dysgalactiae* (DSMZ 20662), *Streptococcus uberis* (DSMZ 20569), *Staphylococcus aureus* (ATCC 6538) and *Staphylococcus epidermidis* (DSMZ 20044).

The minimum inhibitory concentration (MIC) and the minimum bactericidal concentration (MBC) of EOs were evaluated with the microbroth dilution assay using 96-well microplates, as described by the European Committee on Antimicrobial Susceptibility Testing (EUCAST) international guidelines [80], slightly modified.

The MIC is defined as the lowest concentration that completely inhibits the growth of a given organism compared with the growth in the negative control (substance-free), while the MBC is defined as the lowest concentration resulting in the death of 99.9% or more of the initial inoculum [81].

The growth medium used for both *S. aureus* and *S. epidermidis* was Mueller Hinton (MH, MERCK, Darmstadt, Germany), while the medium used for *S. agalactiae*, *S. dysgalactiae*, and *S. uberis* was Brain Heart Infusion (BHI, Sigma-Aldrich, Saint Louis, MO, USA).

Bacterial cultures, grown overnight in MH broth or BHI broth, were diluted to McFarland standard No 0.5, and adjusted in order to have 5 × 10^5^ CFU/mL of bacterial suspension in each well. The MIC test was performed on a 96-well microtiter plate: aliquots of 50 μL of EO emulsion, prepared in MH or BHI medium, were added to 50 μL of bacterial suspension made in the same medium. EO’s effectiveness was tested using two-fold scalar dilutions of concentrations between 3.125% (31.25 mL/L) and 0.001% *v*/*v* (0.01 mL/L).

MH broth, added with and without 9.38% Alkamus, was used as positive control. The microplates were sealed with an adhesive film (to avoid dispersion of the EO more volatile components) and incubated at 37 °C for 24 h. After incubation, the bacterial growth was evaluated spectrophotometrically at 620 nm (Multiskan EX, Thermo Fisher Scientific Oy, Vaanta, Finland).

To assess MBC values, 10 μL were taken from each well showing no microbial growth and seeded onto growth medium MH agars. After 24 h of incubation, CFUs were counted to assess cell viability.

Each assay was performed in triplicate and repeated twice.

### 3.5. Inhibition of Biofilm Growth (Biomass Production) and Development

*S. aureus* ATCC 6538 and *S. epidermidis* DSMZ 20044 strains were used for determining inhibition of bacterial biofilm. Brain Heart Infusion (BHI) agar plates were prepared and used for overnight culture of strains at 37 °C without shaking.

Freshly prepared BHI broth, in 10 mL sterile tubes, was inoculated with bacteria from the BHI agar plates and incubated overnight for 24 h at 37 °C, with shaking. The overnight bacterial culture was then diluted, after the culture media was used to blank, with sterile distilled water to 0.5 McFarland standard (1.5 × 10^8^ CFU/ml) and then further dilute 1:200 in the culture media BHI broth to obtain an inoculum of 0.75 × 10^6^ CFU/mL.

To study the activity of the essential oils and the two blends on the biofilm formation, natural compounds were evaluated at a concentration range from 0.78% *v*/*v* to 0.195% *v*/*v*.

#### 3.5.1. Effect on Bacterial Adhesion (EBA)

The modified protocol described by Kırmusaoğlu and Kaşıkçı (2020) [82] was followed.

An aliquot of 100 µL of diluted culture were added to the wells of individually labelled flat-bottomed 96-well microtitre plates, apart from the wells designed for blanks (media blank, samples and positive control blank). In total, 100 µL of samples (essential oils and blends) and control were added immediately after to determine the inhibition of cell attachment (EBA). Control wells were included in the plate: negative control (culture + media (BHI)), positive control (culture +BHI+ antibiotic), sample control (sample + BHI), antibiotic control (antibiotic + BHI) and media control (BHI) for each test batch. Gentamicin (Sigma-Aldrich, Saint Louis, MO, USA) was used as positive control, at concentrations from 1 mg/mL to 0.25 mg/mL.

Plates were then covered with an adhesive film (to avoid dispersion of the EO more volatile components) and incubated for 24 h at 37 °C, without shaking, to allow cell attachment and biofilm development. The modified crystal violet staining method, described below (Section 3.5.3), was used to quantify the biomass.

#### 3.5.2. Effect on Pre-Formed Biofilm (EPB)

The ability of the EOs to prevent further biofilm development or destruction of pre-formed biofilms was explored using the protocol described by O’Toole and Kolter (1998) [83] and modified by Sandasi et al. (2010) [84]. In total, 100 µL of diluted culture of *S. aureus* or *S. epidermidis* were added to an individually labelled flat-bottomed 96-well microtiter plate and incubated at 37 °C for 24 h (irreversible attachment phase) without agitation for the formation of a multilayer biofilm. After 24 h (EPB), 100 µL aliquots of essential oils and of blends GL and BR, at the different concentrations, were then added to the wells and the plates were sealed and additionally incubated at 37 °C for 24 h. The controls were the same as the previous paragraph: negative control, positive control, sample control, antibiotic control and media control.

Biofilm biomass was assessed using the modified crystal violet staining assay as described below.

#### 3.5.3. Crystal Violet Staining (CVS) Procedure

The attached biofilm mass was indirectly assessed by the modified crystal violet assay [84]. After incubating with the treatment, the wells were carefully emptied and gently washed three times with sterile distilled water to remove any unattached cells. Then the plates were air-dried and subsequently oven-dried at 45 °C for 60 min to fix the adherent cells. The sessile cells were then stained with 100 µL of 0.1% crystal violet solution (CV, Sigma-Aldrich, Saint Louis, MO, USA) for 15 min at room temperature while covered with the microtitre lid. The plate was gently washed five times with sterile distilled water to remove any excess stain and unabsorbed stain.

Thereafter, the biofilm biomass was assessed semi-quantitatively by re-solubilizing the crystal violet stain bound to the adherent cells with 150 µL of 100% ethanol. The absorbance of the destaining solution was read at 570 nm using a microplate reader (Infinite F50, TECAN, Salzburg, Austria). The mean optical density (OD_570nm_) of the sample was determined, and the percentage inhibition of biofilm was calculated using the equation below:

(1) $$\%\;\mathrm{Inhibition}=\frac{({\mathrm{OD}}_{\mathrm{negative}\;\mathrm{control}}\;-{\mathrm{OD}}_{\mathrm{media}\;\mathrm{control}})\;-({\mathrm{OD}}_{\mathrm{test}}-{\mathrm{OD}}_{\mathrm{extract}\;\mathrm{control}})\;}{\left({\mathrm{OD}}_{\mathrm{negative}\;\mathrm{control}}\;-{\mathrm{OD}}_{\mathrm{media}\;\mathrm{control}}\right)}*100$$

Both tests, EBA and EPB, were conducted in triplicate and repeated twice.

Microsoft Excel was used to plot sample concentrations against percentage inhibitions.

## 4. Conclusions

This research was conducted to identify useful tools for the prevention and control of bovine mastitis that would not increase the already widespread phenomenon of antibiotic resistance.

The essential oils chosen and tested were used alone and in combination with each other within two blends to evaluate possible synergistic effects. The antimicrobial and anti-biofilm activities of EOs against *S. epidermidis* and *S. aureus*, the predominant contagious pathogens related to bovine mastitis, were evaluated and our results showed good bacterial and biofilm inhibiting activities of the investigated EOs, especially of *Origanum vulgare* and *Cinnamomum verum*.

The study highlights the importance of evaluating natural products to strengthen the hypothesis of safe EO applications for reducing bacterial contamination as effective antibiotic alternatives in complementary therapy. Furthermore, treatment failure due to antimicrobial resistance could be limited by using combinations of the available antibiotics and other natural compound such as EOs.

## Figures and Tables

**Table 1:** Minimum Inhibitory Concentration (MIC) and Minimum Bactericidal Concentration (MBC) of essential oils and blends against all the tested strains. The MIC and MBC concentrations are given as percentage % *v*/*v* (μL/mL); in bold, the lower MIC and MBC values.

	*S. agalactiae*		*S. dysgalactiae*		*S. uberis*		*S. aureus*		*S. epidermidis*	
Essential Oil	MIC	MBC	MIC	MBC	MIC	MBC	MIC	MBC	MIC	MBC
*Origanum vulgare*	**0.390**	>3.125	**0.098**	**0.195**	**0.195**	**0.390**	**0.390**	>3.125	>3.125	>3.125
*Melaleuca alternifolia*	1.560	>3.125	**0.780**	1.560	**0.780**	1.560	>3.125	>3.125	>3.125	>3.125
*Melaleuca leucadendra*	1.560	>3.125	**0.780**	1.560	3.125	>3.125	>3.125	>3.125	>3.125	>3.125
*Eucalyptus globulus*	>3.125	>3.125	1.560	3.125	>3.125	>3.125	>3.125	>3.125	>3.125	>3.125
*Mentha × piperita*	**0.780**	>3.125	**0.195**	**0.780**	**0.195**	**0.780**	>3.125	>3.125	>3.125	>3.125
*Lavandula angustifolia*	**0.780**	>3.125	**0.195**	**0.390**	**0.195**	**0.195**	>3.125	>3.125	>3.125	>3.125
*Lavandula × intermedia*	**0.780**	>3.125	**0.195**	**0.390**	1.560	3.125	>3.125	>3.125	>3.125	>3.125
*Cinnamomum verum*	**0.050**	**0.098**	**0.098**	**0.195**	**0.001**	**0.001**	**0.195**	**0.390**	**0.098**	**0.195**
*Salvia rosmarinus*	1.560	>3.125	**0.195**	**0.780**	3.125	>3.125	>3.125	>3.125	>3.125	>3.125
*Citrus limon*	3.125	>3.125	3.125	>3.125	>3.125	>3.125	>3.125	>3.125	>3.125	>3.125
*Thymus vulgaris*	**0.390**	>3.125	**0.195**	**0.390**	0.195	**0.780**	>3.125	>3.125	>3.125	>3.125
Blend GL	**0.780**	>3.125	**0.098**	**0.195**	**0.390**	3.125	>3.125	>3.125	>3.125	>3.125
Blend BR	**0.390**	**0.780**	**0.050**	**0.390**	**0.195**	**0.390**	3.125	>3.125	>3.125	>3.125

## Data Availability

The original contributions presented in this study are included in the article and Supplementary Materials. Further inquiries can be directed to the corresponding authors.

## Supplementary Materials

The following supporting information can be downloaded at: https://www.mdpi.com/article/10.3390/antibiotics15101038/s1, Table S1: Chemical composition of the essential oil of *Origanum vulgare* (LRI: Linear Retention Index); Table S2: Chemical composition of the essential oil of *Eucalyptus globulus* (LRI: Linear Retention Index); Table S3: Chemical composition of the essential oil of *Melaleuca alternifolia* (LRI: Linear Retention Index); Table S4: Chemical composition of the essential oil of *Melaleuca leucadendra* (LRI: Linear Retention Index); Table S5: Chemical composition of the essential oil of *Mentha × piperita* (LRI: Linear Retention Index); Table S6: Chemical composition of the essential oil of *Lavandula angustifolia* (LRI: Linear Retention Index); Table S7: Chemical composition of the essential oil of *Lavandula × intermedia* (LRI: Linear Retention Index); Table S8: Chemical composition of the essential oil of *Cinnamomum verum* (LRI: Linear Retention Index); Table S9: Chemical composition of the essential oil of *Salvia rosmarinus* (LRI: Linear Retention Index); Table S10: Chemical composition of the essential oil of *Citrus limon* (LRI: Linear Retention Index); Table S11: Chemical composition of the essential oil of *Thymus vulgaris* (LRI: Linear Retention Index); Table S12: Chemical composition of the essential oil of the Blend GL (LRI: Linear Retention Index); Table S13: Chemical composition of the essential oil of the Blend BR (LRI: Linear Retention Index); Figure S1: Percentage (%) of biofilm inhibition of three different concentrations (% *v*/*v*, μL/mL) of *Lavandula angustifolia* EO on *S. aureus*. EBA = Effect on bacterial adhesion, EPB = Effect on pre-formed biofilm. Vertical bars indicate ± standard deviation; Figure S2: Percentage (%) of biofilm inhibition of three different concentrations (% *v*/*v*, μL/mL) of *Lavandula angustifolia* EO on *S. epidermidis*. EBA = Effect on bacterial adhesion, EPB = Effect on pre-formed biofilm. Vertical bars indicate ± standard deviation; Figure S3: Percentage (%) of biofilm inhibition of three different concentrations (% *v*/*v*, μL/mL) of *Lavandula × intermedia* EO on *S. aureus*. EBA = Effect on bacterial adhesion, EPB = Effect on pre-formed biofilm. Vertical bars indicate ± standard deviation; Figure S4: Percentage (%) of biofilm inhibition of three different concentrations (% *v*/*v*, μL/mL) of *Lavandula × intermedia* EO on *S. epidermidis*. EBA = Effect on bacterial adhesion, EPB = Effect on pre-formed biofilm. Vertical bars indicate ± standard deviation; Figure S5: Percentage (%) of biofilm inhibition of three different concentrations (% *v*/*v*, μL/mL) of *Melaleuca alternifolia* EO on *S. aureus*. EBA = Effect on bacterial adhesion, EPB = Effect on pre-formed biofilm. Vertical bars indicate ± standard deviation; Figure S6: Percentage (%) of biofilm inhibition of three different concentrations (% *v*/*v*, μL/mL) of *Melaleuca alternifolia* EO on *S. epidermidis*. EBA = Effect on bacterial adhesion, EPB = Effect on pre-formed biofilm. Vertical bars indicate ± standard deviation; Figure S7: Percentage (%) of biofilm inhibition of three different concentrations (% *v*/*v*, μL/mL) of *Mentha × piperita* EO on *S. aureus*. EBA = Effect on bacterial adhesion, EPB = Effect on pre-formed biofilm. Vertical bars indicate ± standard deviation; Figure S8: Percentage (%) of biofilm inhibition of three different concentrations (% *v*/*v*, μL/mL) of *Mentha × piperita* EO on *S. epidermidis*. EBA = Effect on bacterial adhesion, EPB = Effect on pre-formed biofilm. Vertical bars indicate ± standard deviation; Figure S9: Percentage (%) of biofilm inhibition of three different concentrations (% *v*/*v*, μL/mL) of *Origanum vulgare* EO on *S. aureus*. EBA = Effect on bacterial adhesion, EPB = Effect on pre-formed biofilm. Vertical bars indicate ± standard deviation; Figure S10: Percentage (%) of biofilm inhibition of three different concentrations (% *v*/*v*, μL/mL) of *Origanum vulgare* EO on *S. epidermidis*. EBA = Effect on bacterial adhesion, EPB = Effect on pre-formed biofilm. Vertical bars indicate ± standard deviation; Figure S11: Percentage (%) of biofilm inhibition of three different concentrations (% *v*/*v*, μL/mL) of *Cinnamomum verum* EO on *S. aureus*. EBA = Effect on bacterial adhesion, EPB = Effect on pre-formed biofilm. Vertical bars indicate ± standard deviation; Figure S12: Percentage (%) of biofilm inhibition of three different concentrations (% *v*/*v*, μL/mL) of *Cinnamomum verum* EO on *S. epidermidis*. EBA = Effect on bacterial adhesion, EPB = Effect on pre-formed biofilm. Vertical bars indicate ± standard deviation; Figure S13: Percentage (%) of biofilm inhibition of three different concentrations (% *v*/*v*, μL/mL) of *Thymus vulgaris* EO on *S. aureus*. EBA = Effect on bacterial adhesion, EPB = Effect on pre-formed biofilm. Vertical bars indicate ± standard deviation; Figure S14: Percentage (%) of biofilm inhibition of three different concentrations (% *v*/*v*, μL/mL) of *Thymus vulgaris* EO on *S. epidermidis*. EBA = Effect on bacterial adhesion, EPB = Effect on pre-formed biofilm. Vertical bars indicate ± standard deviation; Figure S15: Percentage (%) of biofilm inhibition of three different concentrations (mg/mL) of Gentamicin on *S. aureus*. EBA = Effect on bacterial adhesion, EPB = Effect on pre-formed biofilm. Vertical bars indicate ± standard deviation; Figure S16: Percentage (%) of biofilm inhibition of three different concentrations (mg/mL) of Gentamicin on *S. epidermidis*. EBA = Effect on bacterial adhesion, EPB = Effect on pre-formed biofilm. Vertical bars indicate ± standard deviation; Figure S17: Percentage (%) of biofilm inhibition of three different concentrations (% *v*/*v*, μL/mL) of the blend GL on *S. aureus*. EBA = Effect on bacterial adhesion, EPB = Effect on pre-formed biofilm. Vertical bars indicate ± standard deviation; Figure S18: Percentage (%) of biofilm inhibition of three different concentrations (% *v*/*v*, μL/mL) of the blend GL on *S. epidermidis*. EBA = Effect on bacterial adhesion, EPB = Effect on pre-formed biofilm. Vertical bars indicate ± standard deviation; Figure S19: Percentage (%) of biofilm inhibition of three different concentrations (% *v*/*v*, μL/mL) of the blend BR on *S. aureus*. EBA = Effect on bacterial adhesion, EPB = Effect on pre-formed biofilm. Vertical bars indicate ± standard deviation; Figure S20: Percentage (%) of biofilm inhibition of three different concentrations (% *v*/*v*, μL/mL) of the blend BR on *S. epidermidis*. EBA = Effect on bacterial adhesion, EPB = Effect on pre-formed biofilm. Vertical bars indicate ± standard deviation.

## Abbreviations

The following abbreviations are used in this manuscript:

EO	Essential oil
AMR	Antimicrobial resistance
MIC	Minimum Inhibitory Concentration
MBC	Minimum Bactericidal Concentration
