# Supplementary material for: Antibacterial and Anti-Biofilm In Vitro Activities of Essential Oils Against Bovine Mastitis-Related Pathogens
Source: Antibiotics (Basel). 2026 Oct 19;15(10):1038. doi: 10.3390/antibiotics15101038 (PMC13700684; doi:10.3390/antibiotics15101038)
Supplement: Supplementary file 1 [file antibiotics-15-01038-s001.zip › antibiotics-4515145-supplementary.pdf]

Antibacterial and Anti-Biofilm In Vitro Activities of Essential Oils Against Bovine Mastitis-Related Pathogens

Alice Caneschi [1,*], Donatella Scarafile [2], Monica Modesto [2], Maurizio Scozzoli [1], Paola Mattarelli [2] and Anna Zaghini [3]

[1] SIROE—Italian Society for Research on Essential Oils (Società Italiana per la Ricerca sugli Oli Essenziali), Viale Regina Elena 299, 00161 Roma, Italy; maurizio@greenvet.com
[2] Department of Agricultural and Food Sciences, University of Bologna, Viale G. Fanin 44, 40127 Bologna, Italy; donatella.scarafile2@unibo.it (D.S.); monica.modesto@unibo.it (M.M.); paola.mattarelli@unibo.it (P.M.)
[3] Department of Veterinary Medical Sciences, University of Bologna, Via Tolara di Sopra 50, Ozzano Emilia, 40064 Bologna, Italy; anna.zaghini@unibo.it
* Correspondence: alice@greenvet.com

Supplementary Materials

Table S1. Chemical composition of the essential oil of *Origanum vulgare* (LRI: Linear Retention Index).

Compounds	LRI	Area%
sabinene	973	0,60
myrcene	990	0,11
p cymene	1023	6,67
limonene	1027	0,13
gamma terpinene	1058	8,85
cis sabinene hydrate	1099	0,11
thymol	1295	2,98
carvacrol	1313	79,29

Table S2. Chemical composition of the essential oil of *Eucalyptus globulus* (LRI: Linear Retention Index).

Compounds	LRI	Area%
alpha pinene	931	1,78
sabinene	974	0,28
myrcene	991	0,44
alpha phellandrene	1004	0,39
alpha terpinene	1016	0,11
1,8-cineole	1036	93,10
gamma terpinene	1058	1,45
terpinen-4-ol	1177	0,26
alpha terpineol	1190	0,76
carvacrol	1302	0,87
aromadendrene	1444	0,13

Table S3. Chemical composition of the essential oil of *Melaleuca alternifolia* (LRI: Linear Retention Index).

Compounds	LRI	Area%
alpha pinene	932	4,53
sabinene	971	0,16
beta pinene	974	0,21
alpha phellandrene	1004	0,17
alpha terpinene	1017	8,31
p cymene	1024	3,02
limonene	1028	2,55
1,8-cineole	1030	2,56
trans-ocimene	1048	0,10
gamma terpinene	1060	17,69
cis linalool oxide	1071	0,34
terpinolene	1088	2,60
camphor	1145	0,29
terpinen-4-ol	1184	41,21
alpha terpineol	1194	6,55
myrtenal	1198	0,15
lavandulyl acetate	1294	0,13
carvacrol	1304	3,35
alpha gurjunene	1413	0,24
aromadendrene	1445	2,05
beta selinene	1492	0,19
valencene	1494	0,26
viridiflorene	1501	1,19
caryophyllene oxide	1593	0,48

Table S4. Chemical composition of the essential oil of *Melaleuca leucadendra* (LRI: Linear Retention Index).

Compounds	LRI	Area%
alpha pinene	931	4,47
beta pinene	973	2,56
myrcene	990	2,73
alpha phellandrene	1002	0,17
alpha terpinene	1015	0,10
1,8-cineole	1034	67,85
gamma terpinene	1058	3,98
linalool	1100	2,89
borneol	1164	0,18
isomenthone	1164	0,17
terpinen-4-ol	1177	0,47
alpha terpineol	1192	8,55
myrtenal	1197	1,55
piperitone	1259	0,44
carvacrol	1303	1,65
beta caryophyllene	1424	0,98

Table S5. Chemical composition of the essential oil of *Mentha × piperita* (LRI: Linear Retention Index).

Compounds	LRI	Area%
alpha pinene	931	0,94
sabinene	971	0,42
beta pinene	974	1,39
myrcene	995	0,18
o-cymene	1023	0,16
limonene	1027	2,52
1,8-cineole	1029	6,42
cis linalool oxide	1070	0,22
linalool	1100	0,11
camphor	1145	0,14
menthone	1156	24,71
neomenthol	1165	8,74
lavandulol	1170	0,17
menthol	1178	36,43
p cymen-8-ol	1184	0,39
alpha terpineol	1190	0,74
pulegone	1242	0,40
piperitone	1257	1,47
geranial	1276	0,48
menthyl acetate	1296	4,71
delta elemene	1340	0,18
geranyl acetate	1388	0,48
beta caryophyllene	1424	3,80
aromadendrene	1449	0,14
alpha humulene	1459	0,70
gamma muurolene	1481	0,10
delta cadinene	1528	0,10
caryophyllene oxide	1591	0,28

Table S6. Chemical composition of the essential oil of *Lavandula angustifolia* (LRI: Linear Retention Index).

Compounds	LRI	Area%
alpha pinene	930	0,55
camphene	945	0,51
myrcene	990	1,01
3-octanol	994	0,20
3-carene	1010	0,30
o-cymene	1022	0,59
p cymene	1027	4,21
limonene	1029	1,95
cis-ocimene	1037	0,10
trans-ocimene	1047	0,17
trans linalool oxide	1087	0,22
linalool	1105	37,32
camphor	1143	0,91
citronellal	1153	0,26
menthone	1156	1,23
borneol	1164	0,26
lavandulol	1173	0,83

terpinen-4-ol	1177	1,01
alpha terpineol	1191	2,61
myrtenal	1197	0,45
linalyl acetate	1264	30,64
geranial	1274	0,16
bornyl acetate	1289	1,07
lavandulyl acetate	1294	0,13
carvacrol	1303	2,03
neryl acetate	1367	1,18
beta cubebene	1386	2,21
beta caryophyllene	1425	5,14
alpha humulene	1459	0,31
caryophyllene oxide	1592	1,25

Table S7. Chemical composition of the essential oil of *Lavandula* × *intermedia* (LRI: Linear Retention Index).

Compounds	LRI	Area%
alpha pinene	930	0,46
camphene	944	0,21
sabinene	973	0,48
beta pinene	977	0,12
myrcene	989	0,59
alpha terpinene	1013	0,14
o-cymene	1022	0,16
p cymene	1026	1,28
limonene	1029	6,10
cis-ocimene	1036	0,39
trans-ocimene	1046	0,23
cis linalool oxide	1070	0,15
trans linalool oxide	1086	0,18
linalool	1104	33,33
6 camphenol	1107	0,11
fenchol	1113	0,16
camphor	1144	8,30
borneol	1165	2,18
lavandulol	1168	0,56
terpinen-4-ol	1177	2,48
alpha terpineol	1190	0,61
myrtenal	1193	0,35
pulegone	1246	0,13
linalyl acetate	1264	32,20
bornyl acetate	1293	1,80
carvacrol	1302	0,53
neryl acetate	1367	0,17
beta cubebene	1385	0,33
beta caryophyllene	1424	2,21
alpha humulene	1459	0,87
ar curcumene	1486	0,31
gamma cadinene	1519	0,20
caryophyllene oxide	1591	0,24

Table S8. Chemical composition of the essential oil of *Cinnamomum verum* (LRI: Linear Retention Index).

Compounds	LRI	Area%
limonene	1027	0,67
1,8-cineole	1032	3,97
cis sabinene hydrate	1099	0,12
trans rose oxide	1124	0,21
pulegone	1245	0,19
piperitone	1261	0,12
trans-cinnamaldeide	1278	79,03
carvacrol	1303	0,56
citronellyl acetate	1361	2,60
beta caryophyllene	1424	5,63
trans-cinnamil acetate	1448	1,49
alpha humulene	1459	0,69
caryophyllene oxide	1592	0,22

Table S9. Chemical composition of the essential oil of *Salvia rosmarinus* (LRI: Linear Retention Index).

Compounds	LRI	Area%
alpha thujene	924	0,18
alpha pinene	930	11,39
camphene	945	0,18
beta pinene	973	12,45
myrcene	990	0,19
3-carene	1008	0,11
o-cymene	1022	4,24
1,8-cineole	1029	34,29
gamma terpinene	1056	4,95
linalool	1102	0,41
camphor	1142	19,35
borneol	1164	0,16
alpha terpineol	1189	1,56
myrtenal	1194	0,18
alpha humulene	1458	3,26
caryophyllene oxide	1591	4,04

Table S10. Chemical composition of the essential oil of *Citrus limon* (LRI: Linear Retention Index).

Compounds	LRI	Area%
alpha thujene	924	0,13
alpha pinene	931	8,16
camphene	944	0,14
beta pinene	975	15,20
myrcene	990	3,87
alpha phellandrene	1002	0,25
3-carene	1008	0,15
p cymene	1023	0,24
limonene	1031	57,55
gamma terpinene	1057	4,81
linalool	1099	1,95
camphor	1142	0,70

trans linalool oxide	1189	0,14
pulegone	1244	1,35
geranial	1273	2,52
carvacrol	1301	0,34
geranyl formate	1307	0,13
beta caryophyllene	1422	0,13
beta farnesene	1458	0,22
caryophyllene oxide	1590	0,41

Table S11. Chemical composition of the essential oil of *Thymus vulgaris* (LRI: Linear Retention Index).

Compounds	LRI	Area%
alpha thujene	923	0,80
alpha pinene	929	1,21
camphene	943	1,08
sabinene	972	0,14
myrcene	988	0,69
alpha phellandrene	1002	0,32
alpha terpinene	1014	1,02
p cymene	1023	19,89
limonene	1026	1,12
1,8-cineole	1028	0,20
gamma terpinene	1057	9,47
terpinolene	1085	0,13
linalool	1099	3,71
camphor	1143	0,29
terpinen-4-ol	1176	0,90
p cymen-8-ol	1189	0,16
alpha terpineol	1191	0,55
myrtenal	1197	0,11
geranial	1275	0,89
thymol	1298	46,41
carvacrol	1303	6,17
beta caryophyllene	1422	2,71
alpha humulene	1457	0,25
caryophyllene oxide	1590	0,19

Table S12. Chemical composition of the essential oil of the Blend GL (LRI: Linear Retention Index).

Compounds	LRI	Area%
alpha thujene	924	0,11
alpha pinene	931	2,33
camphene	945	0,15
beta pinene	974	2,76
myrcene	990	8,48
alpha phellandrene	1003	0,14
alpha terpinene	1015	1,10
p cymene	1023	3,38
limonene	1029	16,81
1,8-cineole	1031	8,19
gamma terpinene	1058	5,17
terpinolene	1080	1,70

linalool	1101	5,50
camphor	1142	0,23
borneol	1166	1,69
terpinen-4-ol	1178	5,13
alpha terpineol	1191	2,15
myrtenal	1196	0,30
carvone	1246	0,13
piperitone	1255	1,02
linalyl acetate	1261	3,33
geranial	1276	0,19
thymol	1295	5,45
carvacrol	1306	16,20
neryl acetate	1367	0,12
beta cubebene	1385	0,28
beta caryophyllene	1423	0,84
aromadendrene	1444	0,32
viridiflorene	1499	0,19
caryophyllene oxide	1591	0,25

Table S13. Chemical composition of the essential oil of the Blend BR (LRI: Linear Retention Index).

Compounds	LRI	Area%
alpha thujene	924	0,20
alpha pinene	930	3,27
camphene	944	0,22
beta pinene	973	3,20
myrcene	989	1,28
alpha phellandrene	1002	0,13
alpha terpinene	1014	2,02
p-cymene	1022	5,34
limonene	1026	12,16
1,8-cineole	1028	9,30
gamma terpinene	1056	8,81
terpinolene	1086	0,59
cis sabinene hydrate	1098	1,53
camphor	1141	0,15
borneol	1164	0,41
terpinen-4-ol	1177	9,03
alpha terpineol	1189	2,74
myrtenal	1195	0,24
pulegone	1244	0,23
trans-cinnamaldeide	1272	6,89
thymol	1293	7,74
carvacrol	1303	20,91
beta caryophyllene	1422	0,99
aromadendrene	1442	0,41
viridiflorene	1498	0,25
caryophyllene oxide	1590	0,13

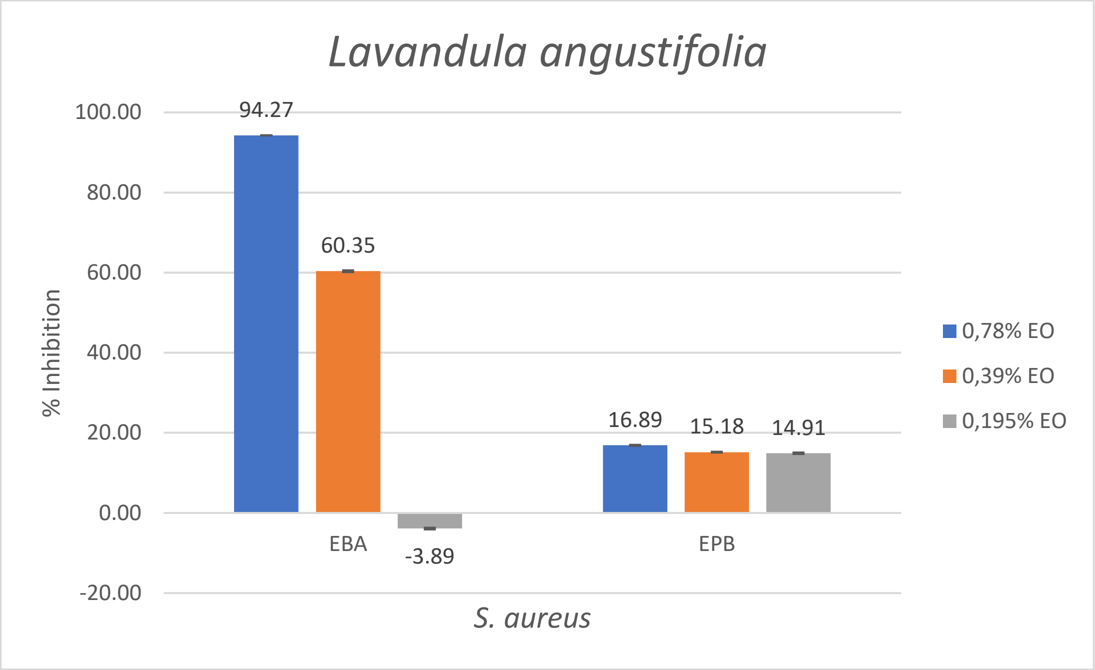

Figure S1. Percentage (%) of biofilm inhibition of three different concentrations (% *v*/*v*, µL/mL) of *Lavandula angustifolia* EO on *S. aureus*. EBA= Effect on bacterial adhesion, EPB= Effect on pre-formed biofilm. Vertical bars indicate ± standard deviation.

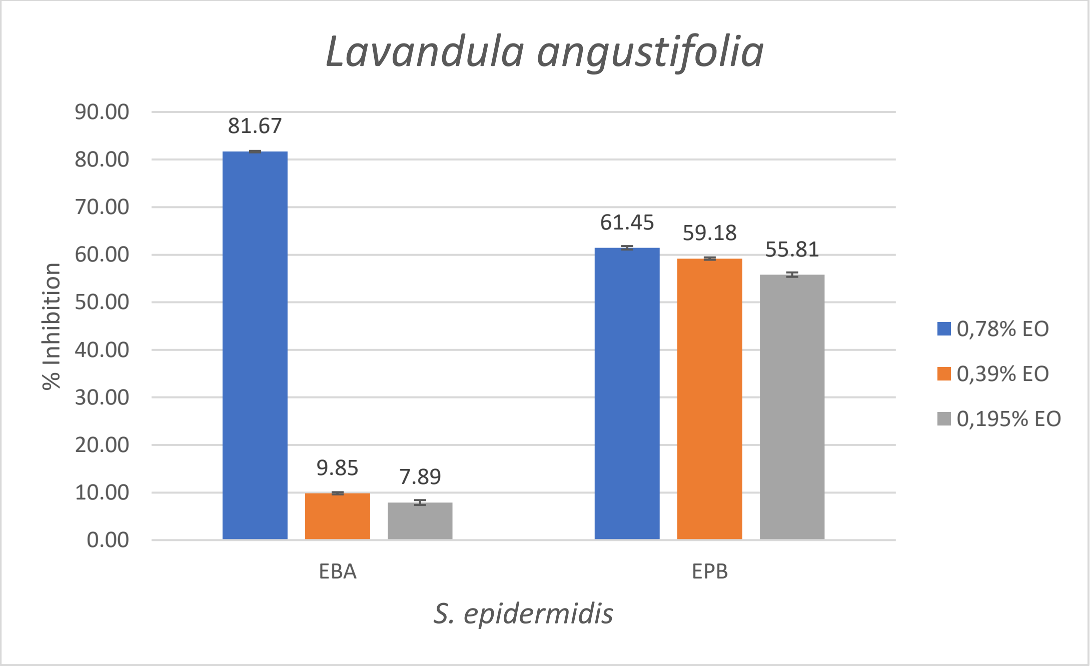

Figure S2. Percentage (%) of biofilm inhibition of three different concentrations (% *v*/*v*, µL/mL) of *Lavandula angustifolia* EO on *S. epidermidis*. EBA= Effect on bacterial adhesion, EPB= Effect on pre-formed biofilm. Vertical bars indicate ± standard deviation.

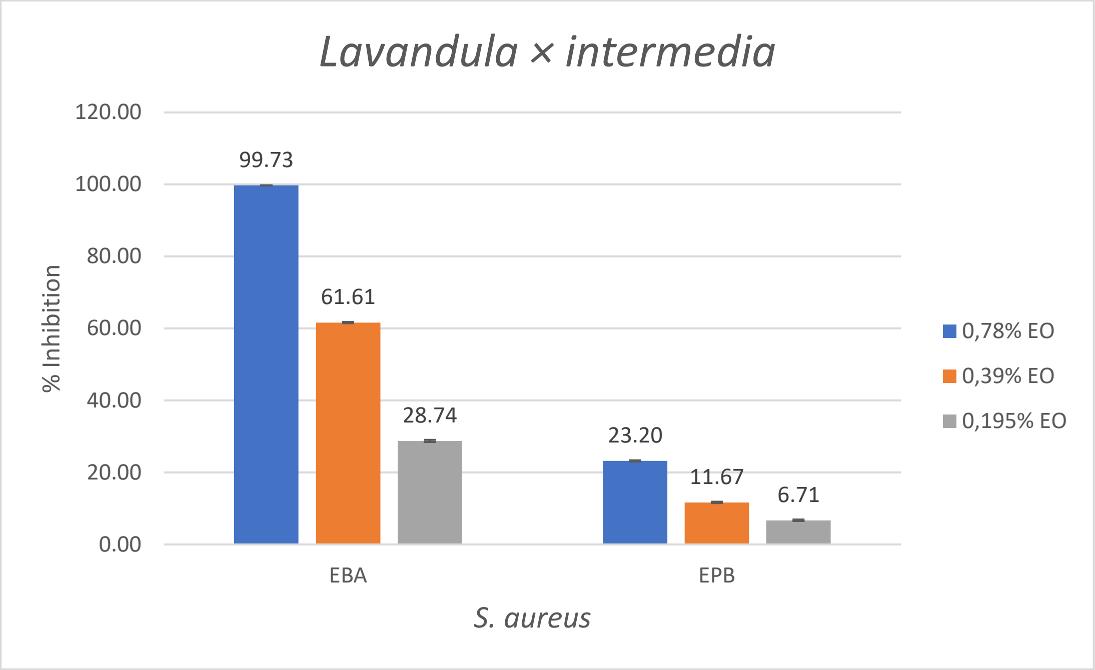

Figure S3. Percentage (%) of biofilm inhibition of three different concentrations (% *v*/*v*, µL/mL) of *Lavandula* × *intermedia* EO on *S. aureus*. EBA= Effect on bacterial adhesion, EPB= Effect on pre-formed biofilm. Vertical bars indicate ± standard deviation.

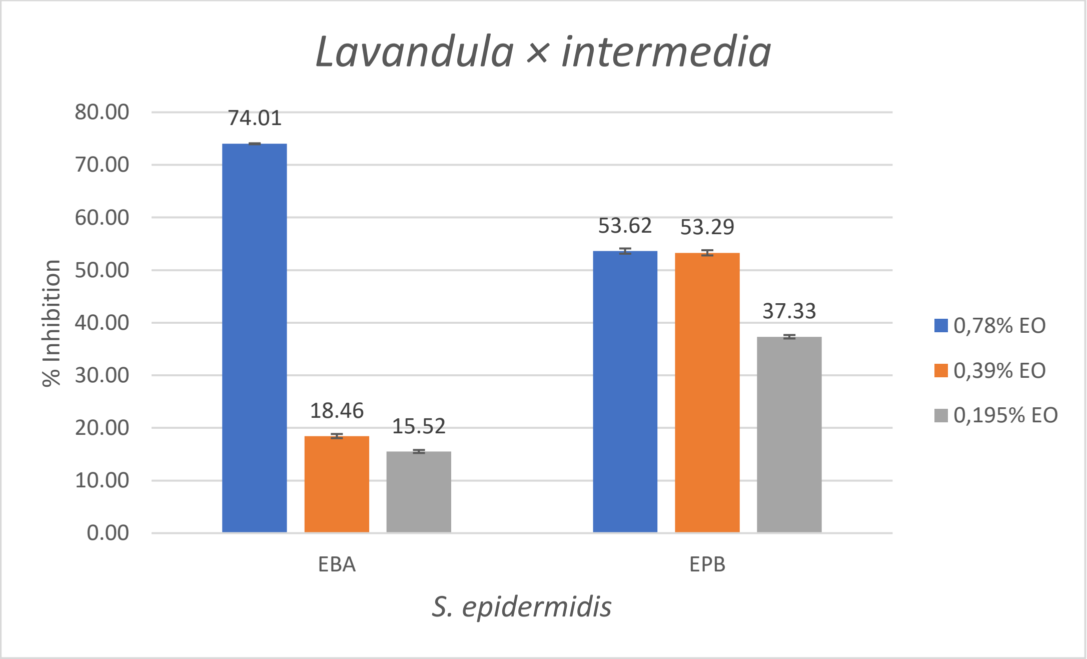

Figure S4. Percentage (%) of biofilm inhibition of three different concentrations (% *v*/*v*, µL/mL) of *Lavandula* × *intermedia* EO on *S. epidermidis*. EBA= Effect on bacterial adhesion, EPB= Effect on pre-formed biofilm. Vertical bars indicate ± standard deviation.

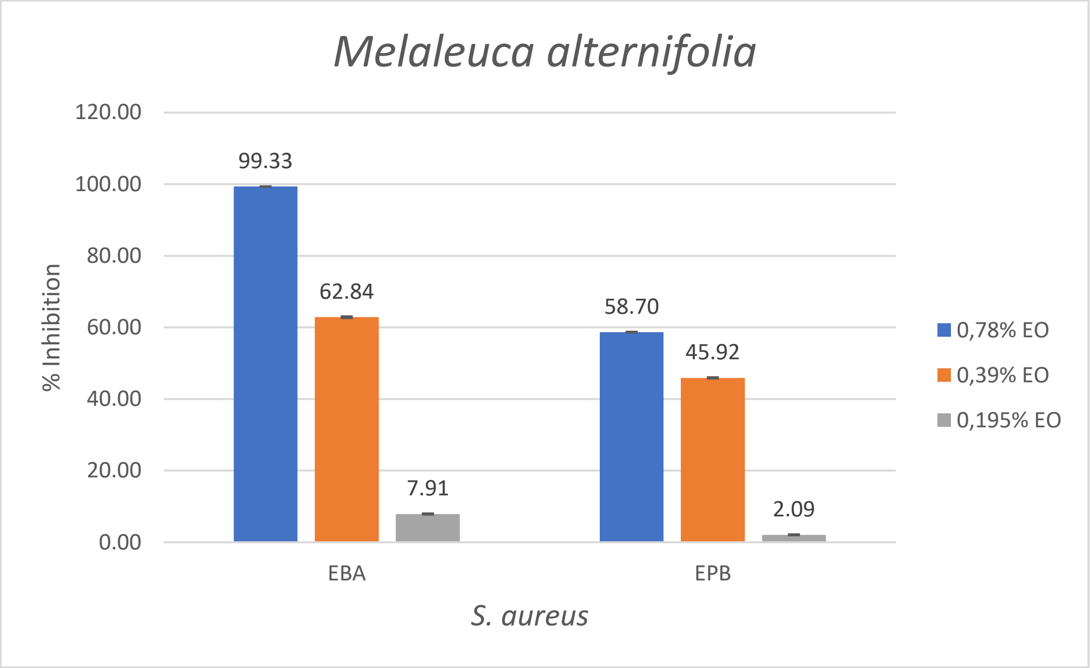

Figure S5. Percentage (%) of biofilm inhibition of three different concentrations (% *v/v*, μL/mL) of *Melaleuca alternifolia* EO on *S. aureus*. EBA= Effect on bacterial adhesion, EPB= Effect on pre-formed biofilm. Vertical bars indicate ± standard deviation.

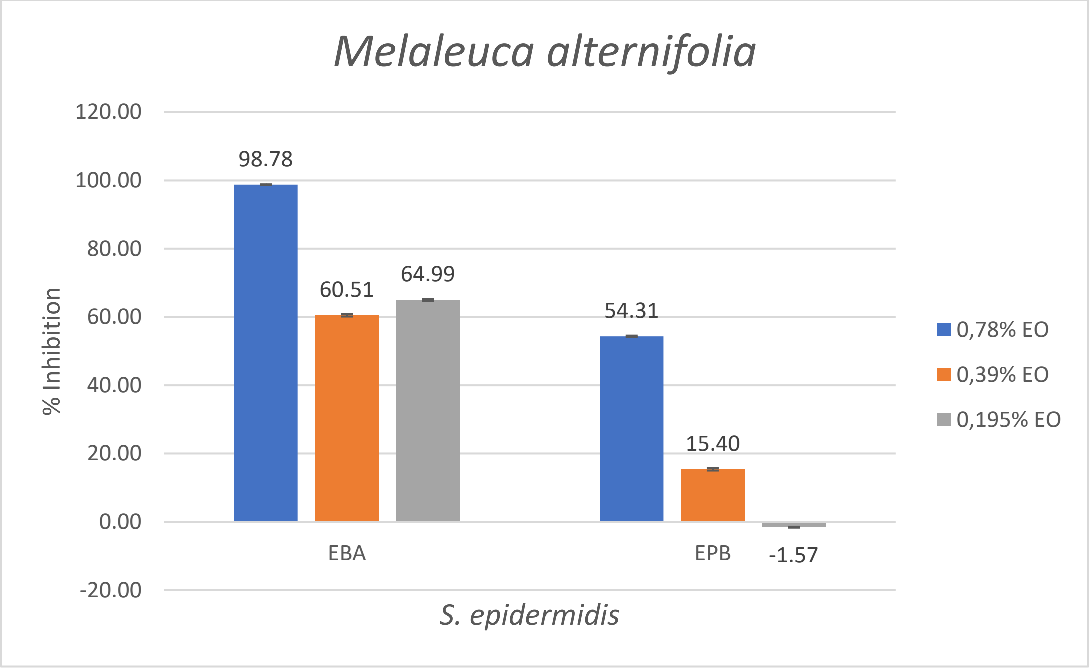

Figure S6. Percentage (%) of biofilm inhibition of three different concentrations (% *v/v*, μL/mL) of *Melaleuca alternifolia* EO on *S. epidermidis*. EBA= Effect on bacterial adhesion, EPB= Effect on pre-formed biofilm. Vertical bars indicate ± standard deviation.

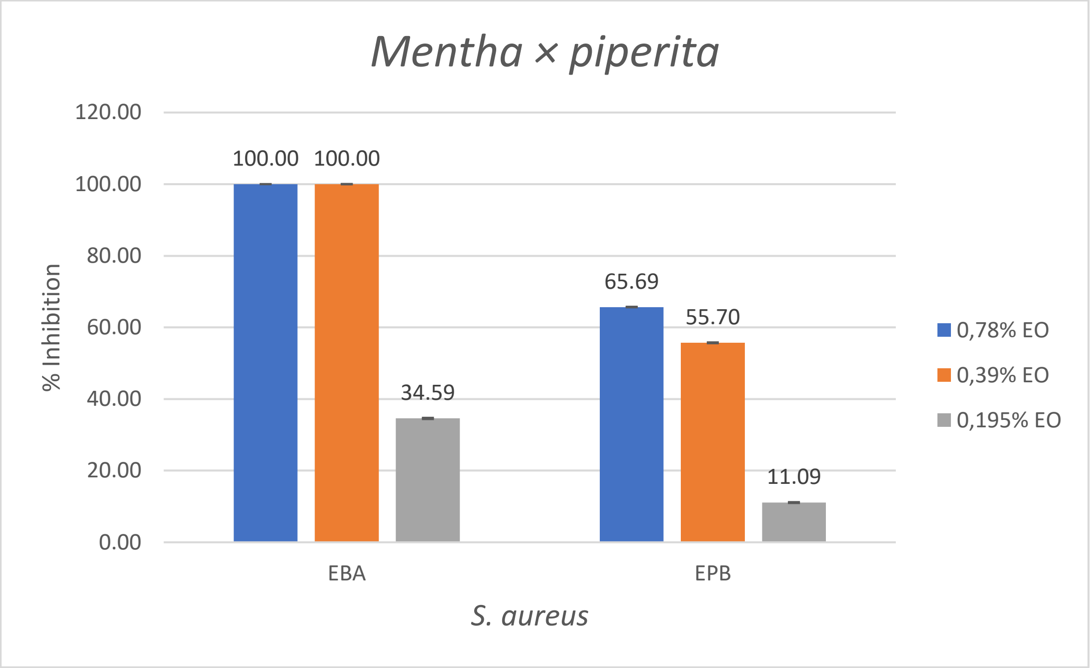

Figure S7. Percentage (%) of biofilm inhibition of three different concentrations (% *v*/*v*, μL/mL) of *Mentha* × *piperita* EO on *S. aureus*. EBA= Effect on bacterial adhesion, EPB= Effect on pre-formed biofilm. Vertical bars indicate ± standard deviation.

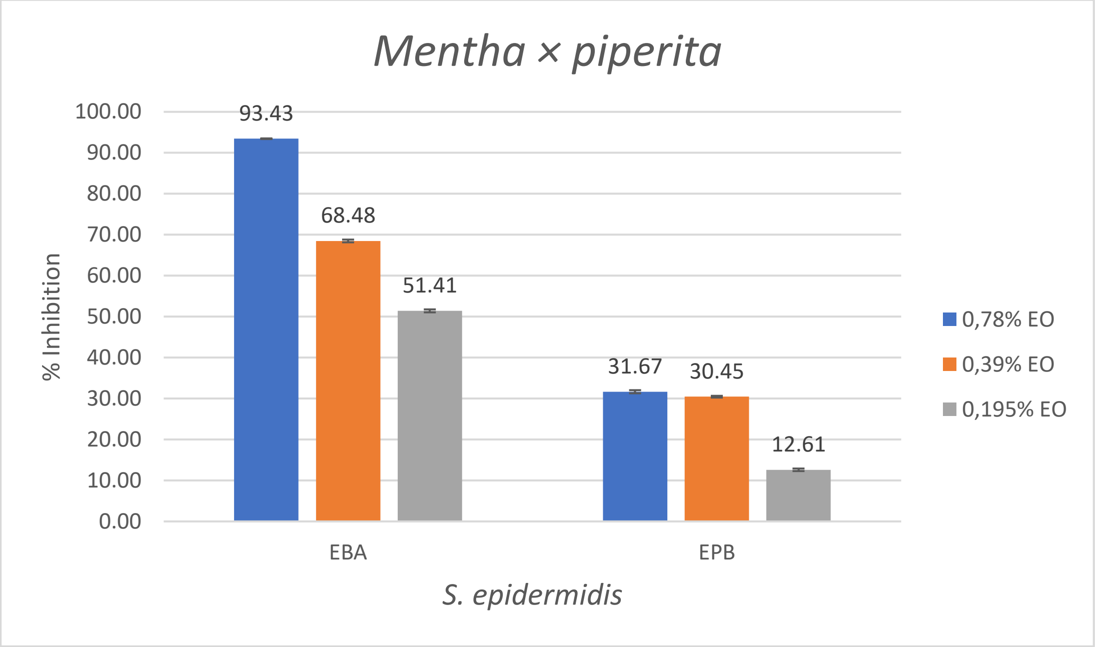

Figure S8. Percentage (%) of biofilm inhibition of three different concentrations (% *v*/*v*, μL/mL) of *Mentha* × *piperita* EO on *S. epidermidis*. EBA= Effect on bacterial adhesion, EPB= Effect on pre-formed biofilm. Vertical bars indicate ± standard deviation.

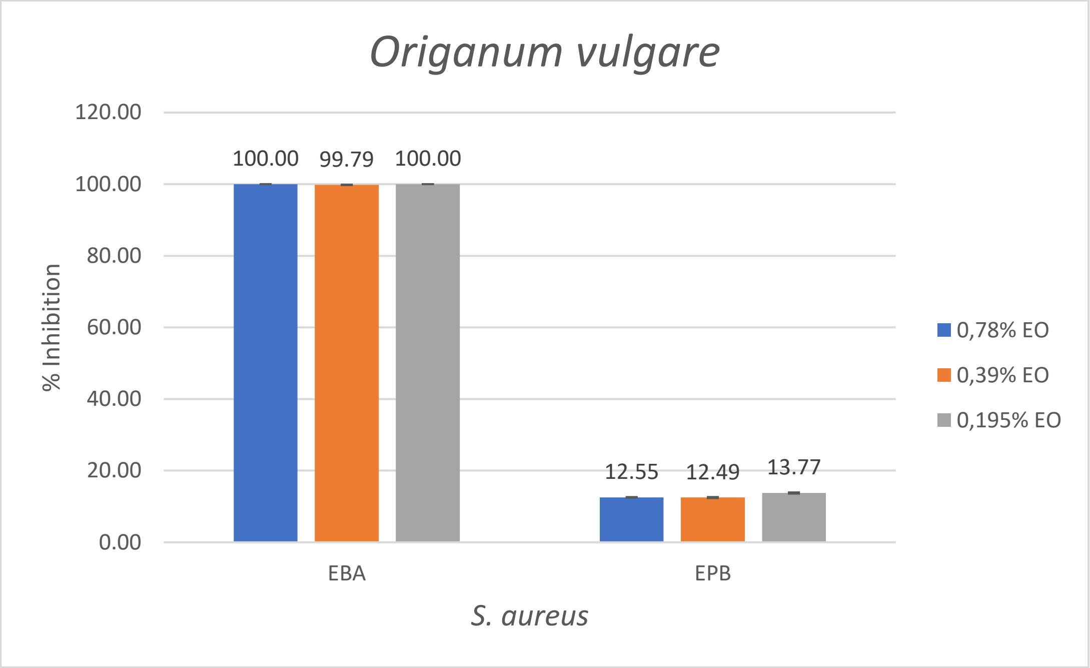

Figure S9. Percentage (%) of biofilm inhibition of three different concentrations (% *v*/*v*, µL/mL) of *Origanum vulgare* EO on *S. aureus*. EBA= Effect on bacterial adhesion, EPB= Effect on pre-formed biofilm. Vertical bars indicate ± standard deviation.

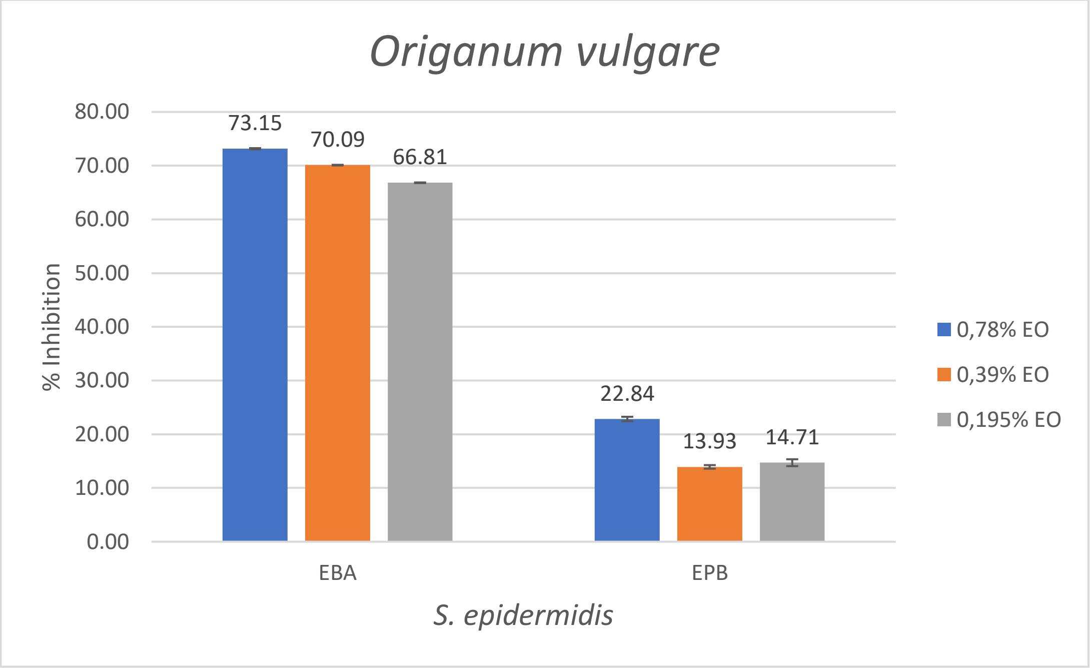

Figure S10. Percentage (%) of biofilm inhibition of three different concentrations (% *v*/*v*, µL/mL) of *Origanum vulgare* EO on *S. epidermidis*. EBA= Effect on bacterial adhesion, EPB= Effect on pre-formed biofilm. Vertical bars indicate ± standard deviation.

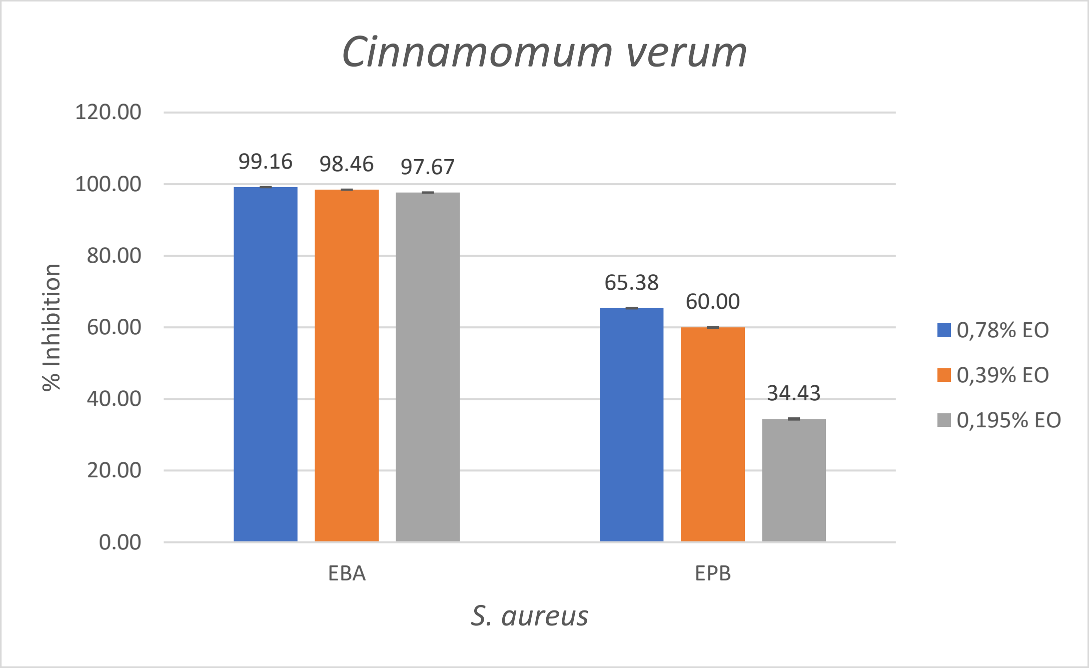

Figure S11. Percentage (%) of biofilm inhibition of three different concentrations (% *v/v*, µL/mL) of *Cinnamomum verum* EO on *S. aureus*. EBA= Effect on bacterial adhesion, EPB= Effect on pre-formed biofilm. Vertical bars indicate ± standard deviation.

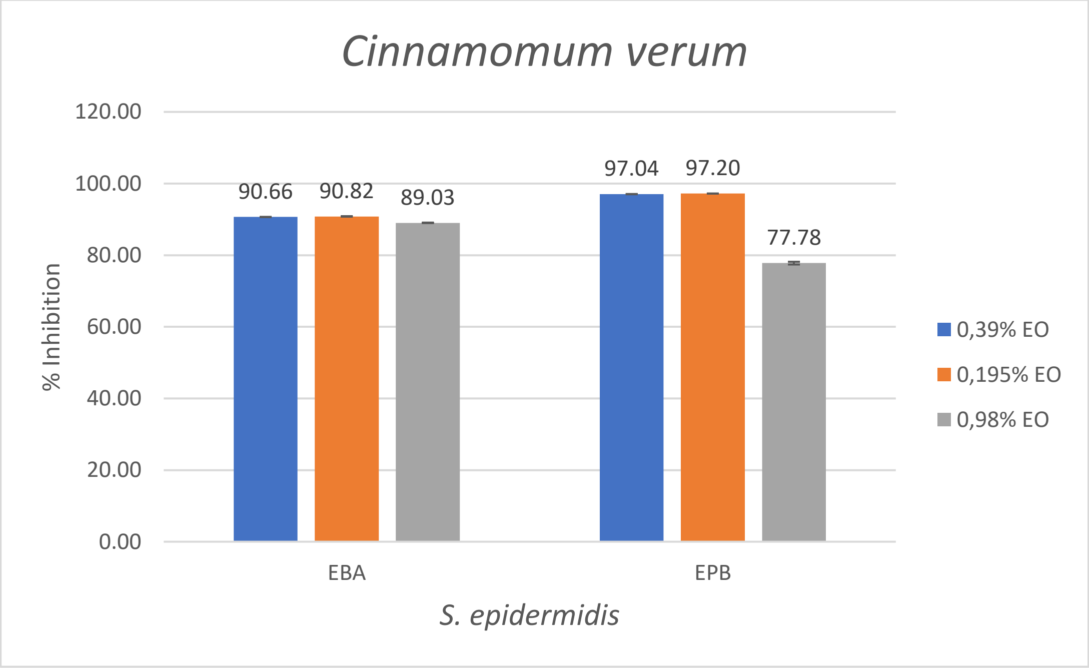

Figure S12. Percentage (%) of biofilm inhibition of three different concentrations (% *v/v*, µL/mL) of *Cinnamomum verum* EO on *S. epidermidis*. EBA= Effect on bacterial adhesion, EPB= Effect on pre-formed biofilm. Vertical bars indicate ± standard deviation.

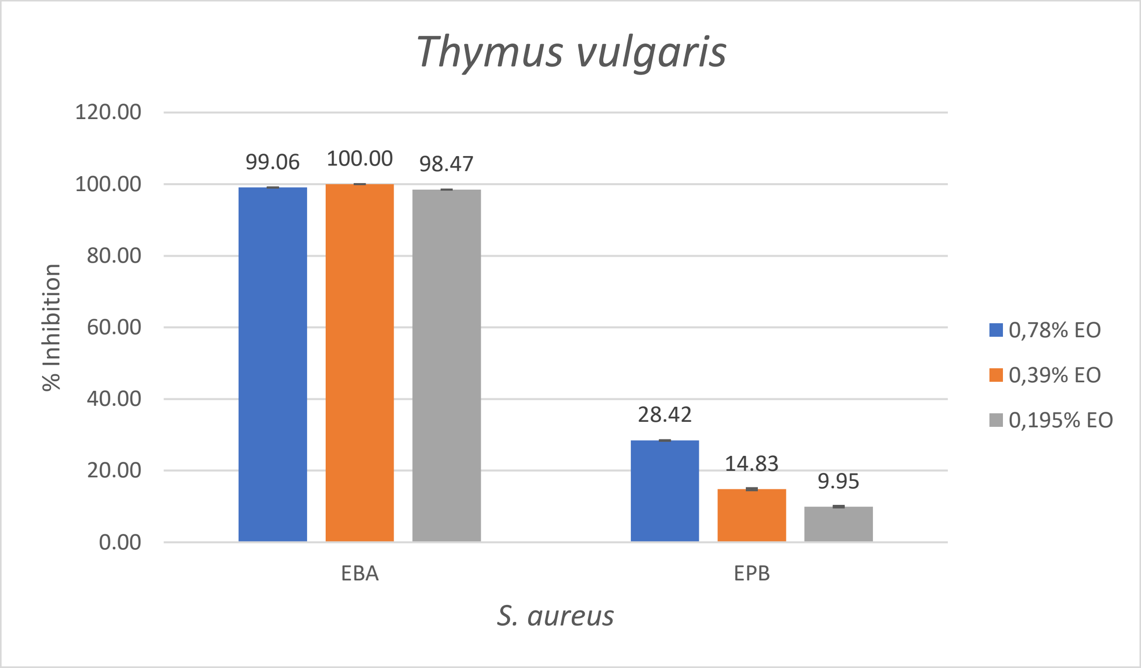

Figure S13. Percentage (%) of biofilm inhibition of three different concentrations (% *v*/*v*, µL/mL) of *Thymus vulgaris* EO on *S. aureus*. EBA= Effect on bacterial adhesion, EPB= Effect on pre-formed biofilm. Vertical bars indicate ± standard deviation.

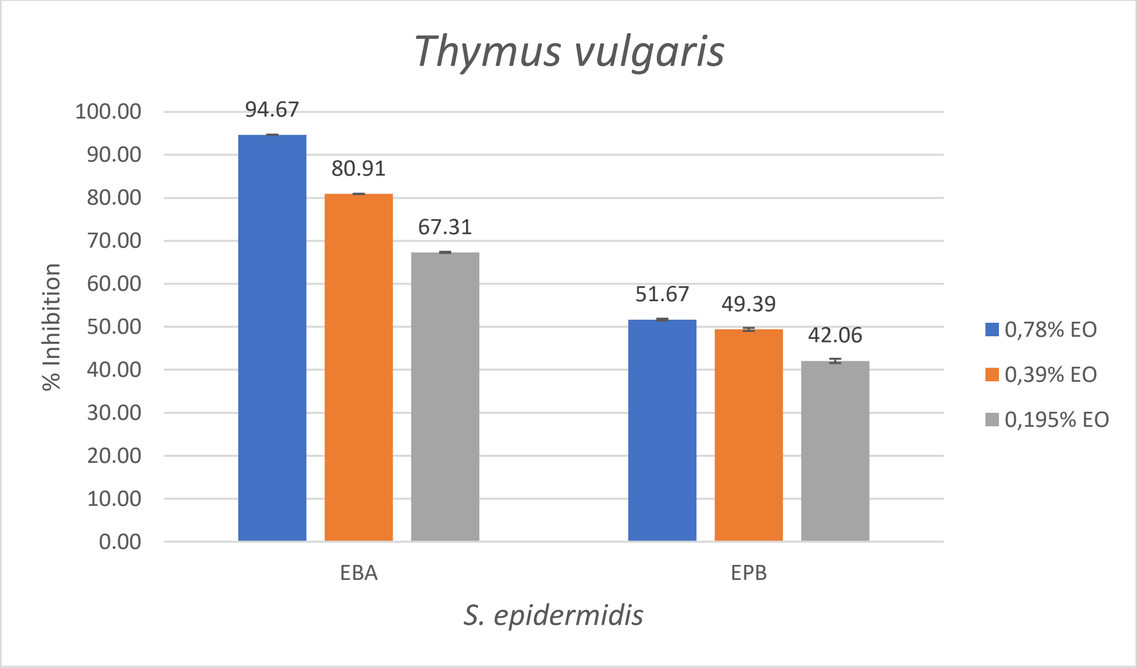

Figure S14. Percentage (%) of biofilm inhibition of three different concentrations (% *v*/*v*, µL/mL) of *Thymus vulgaris* EO on *S. epidermidis*. EBA= Effect on bacterial adhesion, EPB= Effect on pre-formed biofilm. Vertical bars indicate ± standard deviation.

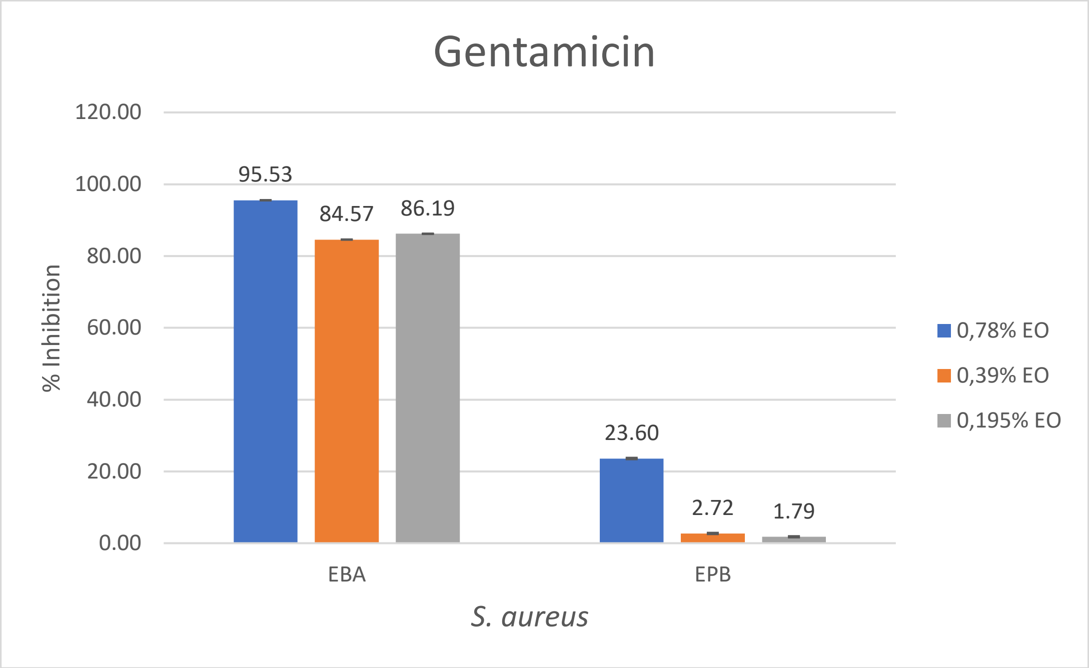

Figure S15. Percentage (%) of biofilm inhibition of three different concentrations (mg/mL) of Gentamicin on *S. aureus*. EBA= Effect on bacterial adhesion, EPB= Effect on pre-formed biofilm. Vertical bars indicate ± standard deviation.

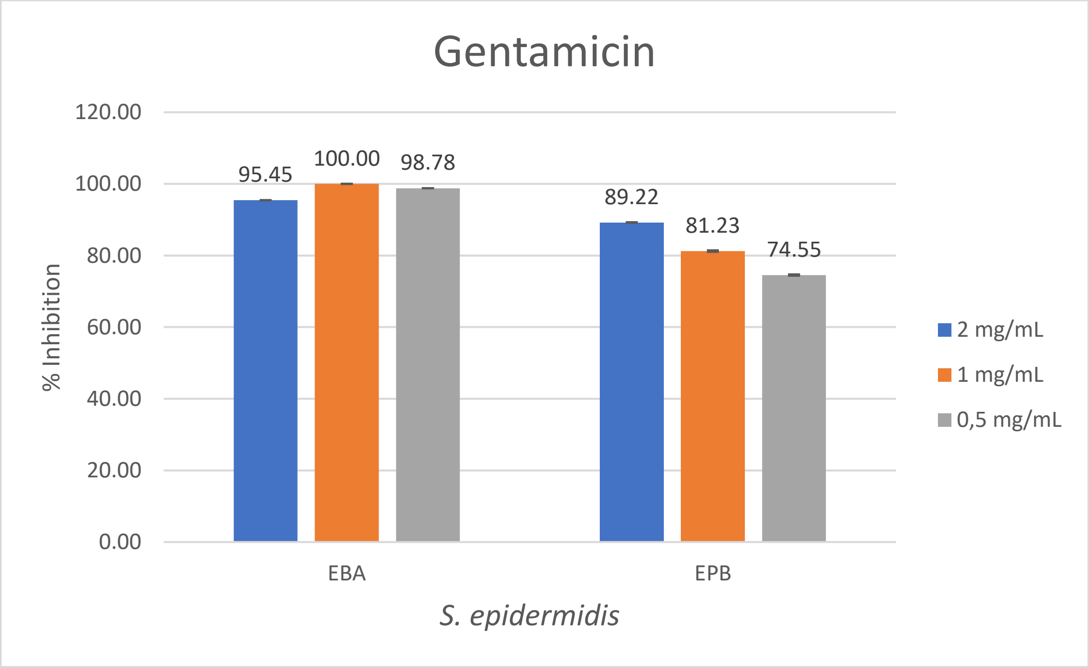

Figure S16. Percentage (%) of biofilm inhibition of three different concentrations (mg/mL) of Gentamicin on *S. epidermidis*. EBA= Effect on bacterial adhesion, EPB= Effect on pre-formed biofilm. Vertical bars indicate ± standard deviation.

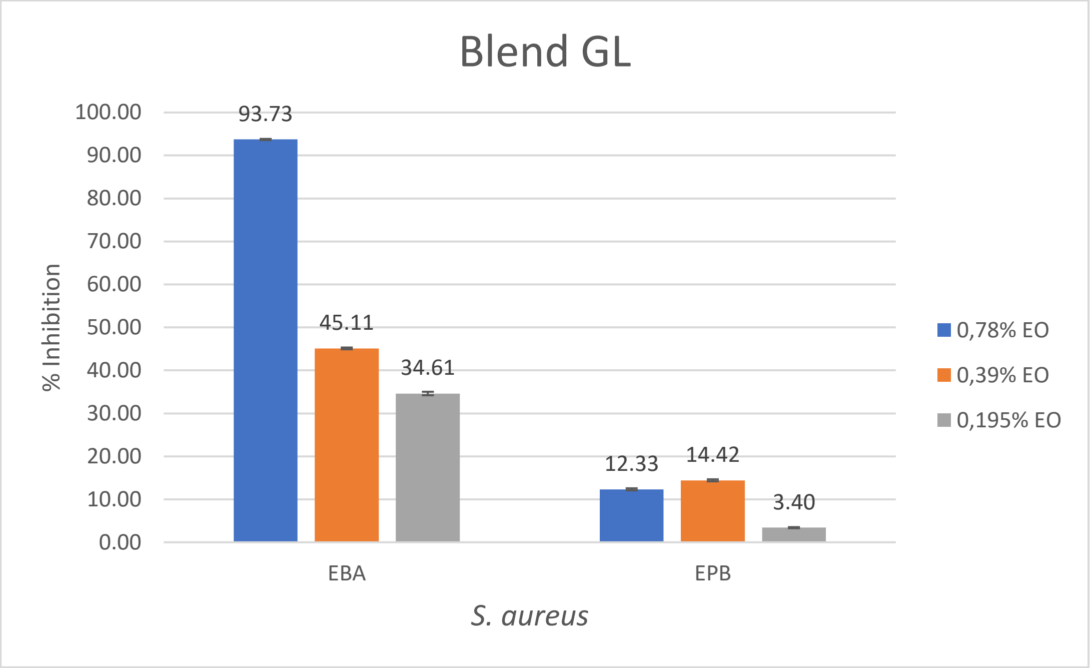

Figure S17. Percentage (%) of biofilm inhibition of three different concentrations (% *v/v*, µL/mL) of the blend GL on *S. aureus*. EBA= Effect on bacterial adhesion, EPB= Effect on pre-formed biofilm. Vertical bars indicate ± standard deviation.

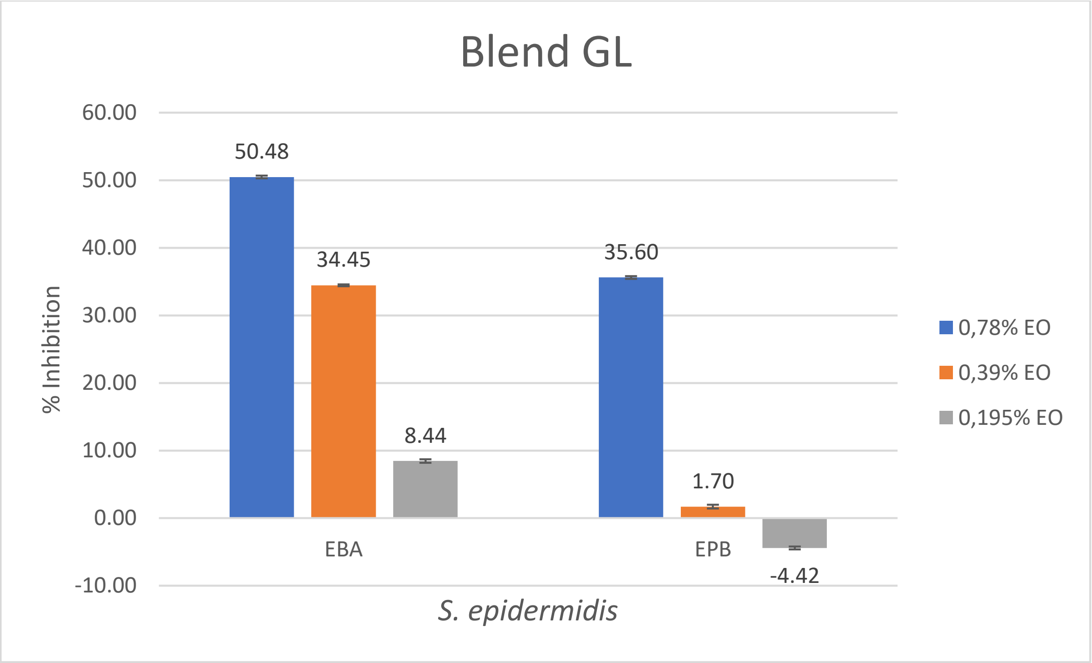

Figure S18. Percentage (%) of biofilm inhibition of three different concentrations (% *v/v*, µL/mL) of the blend GL on *S. epidermidis*. EBA= Effect on bacterial adhesion, EPB= Effect on pre-formed biofilm. Vertical bars indicate ± standard deviation.

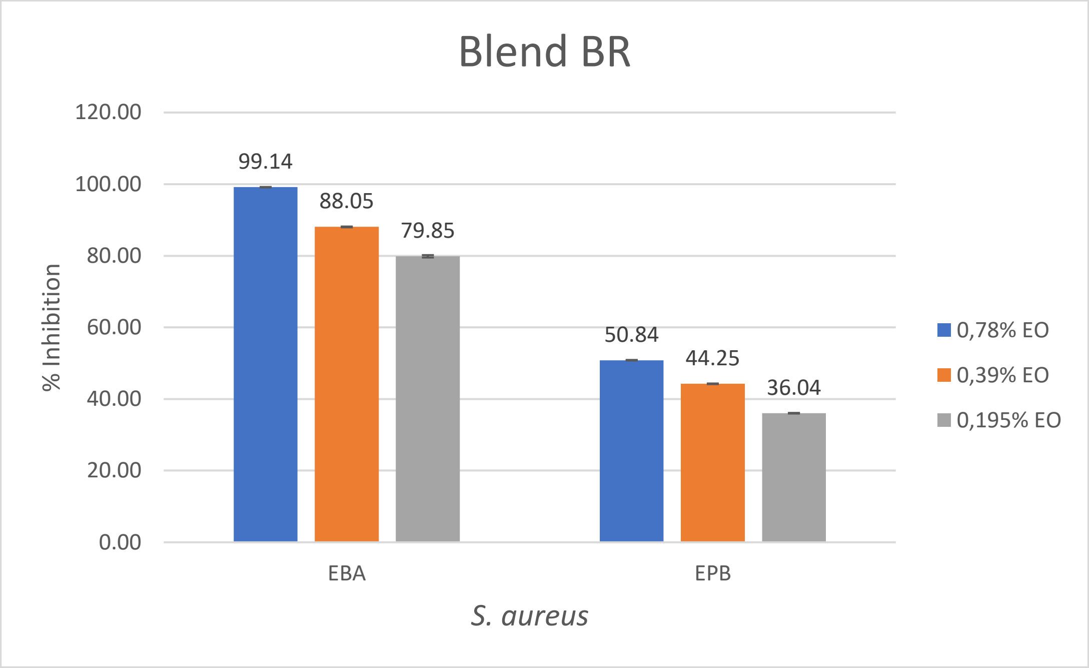

Figure S19. Percentage (%) of biofilm inhibition of three different concentrations (% *v*/*v*, µL/mL) of the blend BR on *S. aureus*. EBA= Effect on bacterial adhesion, EPB= Effect on pre-formed biofilm. Vertical bars indicate ± standard deviation.

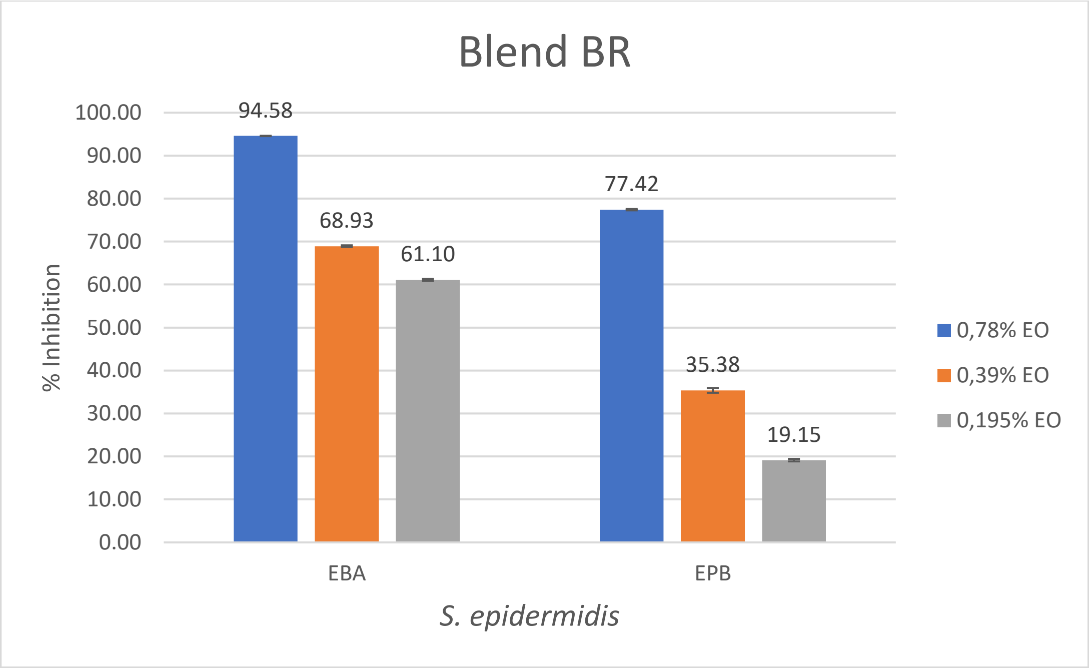

Figure S20. Percentage (%) of biofilm inhibition of three different concentrations (% *v*/*v*, µL/mL) of the blend BR on *S. epidermidis*. EBA= Effect on bacterial adhesion, EPB= Effect on pre-formed biofilm. Vertical bars indicate ± standard deviation.
